# Are the St John’s wort Hyp-1 superstructures different?

**DOI:** 10.1107/S2059798321003740

**Published:** 2021-05-14

**Authors:** Jeffrey J. Lovelace, Gloria E. O. Borgstahl

**Affiliations:** a Eppley Institute for Research in Cancer and Allied Diseases, University of Nebraska Medical Center, 986805 Nebraska Medical Center, Omaha, NE 68198-6805, USA

**Keywords:** modulation, tNCS, supercell, superspace

## Abstract

Two commensurately modulated structures (PDB entries 4n3e and 6sjj) were solved using translational noncrystallographic symmetry (tNCS). The data required the use of large supercells, sevenfold and ninefold, respectively, to properly index the reflections. Commensurately modulated structures can be challenging to solve. Molecular-replacement software such as *Phaser* can detect tNCS and either handle it automatically or, for more challenging situations, allow the user to enter a tNCS vector, which the software then uses to place the components. Although this approach has been successful in solving these types of challenging structures, it does not make it easy to understand the underlying modulation in the structure or how these two structures are related. An alternate view of this problem is that the atoms and associated parameters are following periodic atomic modulation functions (AMFs) in higher dimensional space, and what is being observed in these supercells are the points where these higher dimensional AMFs intersect physical 3D space. In this case, the two 3D structures, with a sevenfold and a ninefold superstructure, seem to be quite different. However, describing those structures within the higher dimensional superspace approach makes a strong case that they are closely related, as they show very similar AMFs and can be described with one unique (3+1)D structure, *i.e.* they are two different 3D intersections of the same (3+1)D structure.

## Abstract 2

Two modulated structures of the Hyp-1–ANS complex with different unit-cell dimensions and contents were analyzed in (3+1)D superspace, revealing that they are very similar if not the same higher-dimensional structure with a slight shift in the **q** vector being responsible for the differences observed in 3D space.

## 1. Introduction   

Commensurately modulated structures are a unique subset of entries in the Protein Data Bank (PDB). The diffraction patterns from these types of structures are distinct in that they consist of strong main reflections with, on average, less intense satellite reflections around the main reflection. Indexing software can usually index the main reflections, but in some cases can have difficulty indexing all of the reflections. For commensurate cases, the indexing software will lock into a solution that is some multiple of the basic unit cell, resulting in a supercell. This supercell will be made of an integer multiple of basic cells along one or more of the unit-cell directions. An excellent approach to solving these structures has successfully been implemented by using the theory behind translational noncrystallographic symmetry (tNCS) to enable molecular-replacement programs (McCoy *et al.*, 2007 ▸; Read & McCoy, 2016 ▸) to arrive at solutions where they may have been unable to do so in the past.

Our laboratory has been working on the incommensurately modulated structure of the profilin–actin complex (Lovelace *et al.*, 2008 ▸; Porta *et al.*, 2011 ▸, 2017 ▸) and has developed a set of superspace-analysis tools to aid us in the analysis of our refinements. Our incommensurate case has proven to be more challenging because there is not a supercell that will predict the satellite reflections, so we have had to use an approximation. Additionally, we have a systematic absence in our reflections when the data are represented as a supercell approximation, which is a characteristic of the underlying modulation that has led to some interesting issues that have required some more innovative solutions during refinement (Lovelace *et al.*, 2018 ▸). The modulated Hyp-1–ANS complex (PDB entry 4n3e, with a sevenfold supercell) and its cousin (PDB entry 6sjj, with a ninefold supercell) provided us with two distinct large commensurately modulated structures (Sliwiak *et al.*, 2015 ▸; Smietanska *et al.*, 2020 ▸) to evaluate our superspace-analysis tools. Dusek *et al.* (2003 ▸) demonstrated for a small molecule that the correctly chosen superspace group can describe the array of crystalline phases (including commensurate and incommensurately modulated phases) observed for sodium carbonate. In superspace, they were able to observe how the atomic modulation functions (AMFs) were similar and determine which atom was responsible for the transition to the incommensurate phase. Using this paper as a template, we suspected that by observing the commensurately modulated superstructures in superspace it should be possible to visualize how these modulated structures are related. The authors of the Hyp-1 structures speculated that there must be some kind of deeper connection between the structures, but pointed out that it is difficult to detect one by comparing the 3D structures. The primary difference in the crystallization conditions was the addition of melatonin for the PDB entry 6jss structure, which leads to a supercell requiring two more basic cells.

### 1.1. tNCS   

**Figure 1:**
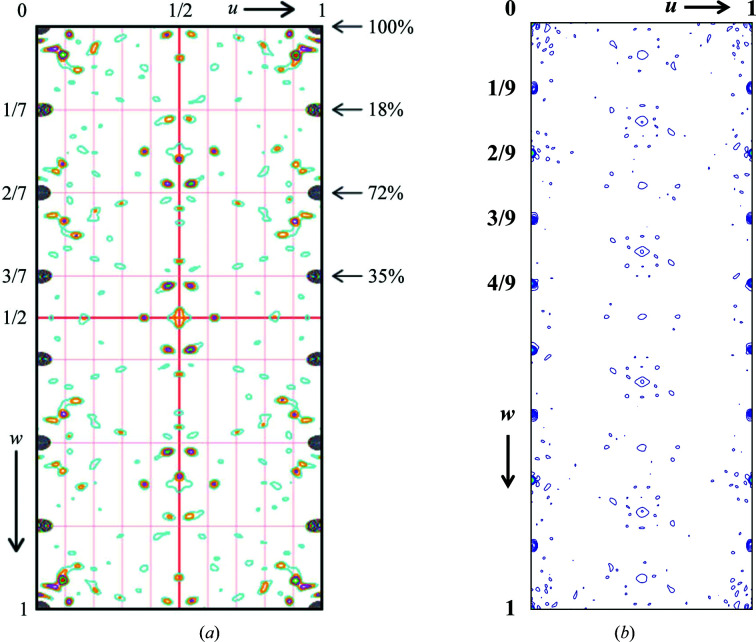
Patterson maps of (*a*) the PDB entry 4n3e data showing the tNCS with a strong peak every 1/7 along the *c* unit-cell direction (*w* Patterson direction; Sliwiak *et al.*, 2015 ▸) and (*b*) the PDB entry 6sjj data showing strong peaks every 1/9 along the *c* unit-cell direction. These strong peaks indicate tNCS in the structures. (*a*) is reproduced from Sliwiak *et al.* (2014 ▸) and (*b*) was generated from the deposited structure factors using Python with *numpy*, *matplotlib* and *gemmi*.

tNCS occurs when the asymmetric unit is made up of a 3D array of identical chains. The chains in this array have slight occupancy, displacement, rotational and/or conformational differences that disrupt the basic cell to basic cell periodic order. If this disruption results in a longer range periodic order, then additional diffraction spots appear, which lead to the need to use a supercell to describe the observed diffraction pattern. This type of case is diagnosed by studying the Patterson map (Patterson, 1935 ▸; Fig. 1 ▸). For PDB entry 4n3e the Patterson map has non-origin strong peaks about every 1/7 of the unit cell (Fig. 1 ▸ *a*), and the Patterson map for PDB entry 6sjj has non-origin strong peaks about every 1/9 of the unit cell (Fig. 1 ▸ *b*); these spacings are consistent with an offset of a whole protein molecule when compared with the unit cell along *c*. This type of protein-sized spacing in the Patterson maps is a strong indication of tNCS. This challenging molecular-replacement problem was solved using tNCS in *Phaser* (McCoy *et al.*, 2007 ▸). Details of the tNCS implementation in *Phaser* and of how it was adapted for PDB entry 4n3e can be found in Sliwiak *et al.* (2014 ▸). Both structures required an innovative multistep approach to arrive at the final solution. Although the structures were solved, it was difficult to use the tNCS data to arrive at an understanding of the underlying modulation. This analysis was complicated by the PDB entry 6sjj structure, which should be related to the PDB entry 4n3e structure but appeared to be unique, requiring an additional two basic cells in the supercell. We decided to investigate whether things may become more evident by looking at the results of refinement from the superspace perspective. Visualization of supercells in superspace is not a new idea and has been successfully used to help to solve incommensurately modulated small-molecule crystals (Schönleber & Chapuis, 2004 ▸). These approaches are no longer seen as often in this field due to the ability of *Superflip* (Palatinus & Chapuis, 2007 ▸) to now directly solve these types of structures.

### 1.2. Superspace notation   

When the underlying periodic structure of a crystal is broken by a periodic distortion of the structure, the resulting superposition of periodic structure and periodic distortion will produce a diffraction pattern that may not be periodic but will still have long-range order and produce diffraction spots. This case is known as a modulated structure. These structures can be described by superspace (higher than three dimensions). For this paper, we are using a variation of the crystallographic notation that can easily be extended into higher dimensions (van Smaalen, 2005 ▸, 2007 ▸). Superspace modulations are identified by the number of extra dimensions needed to describe the diffraction pattern: (3+*d*)D, where *d* is the number of extra dimensions. The simplest of these would be (3+1)D. The unit cell is defined by **a**_1_, **a**_2_, …, **a**_*n*_ instead of **a**, **b** and **c**. The reciprocal-space vectors are 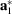, 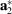, …, 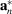 instead of 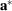, 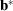 and 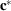. Distances are measured as the values **x**_1_, **x**_2_, …, **x**_*n*_ as opposed to **x**, **y** and **z**. Reflections are labelled *h*_1_, *h*_2_, …, *h_n_* instead of *h*, *k*, *l*, *m*.

### 1.3. Superspace   

**Figure 2:**
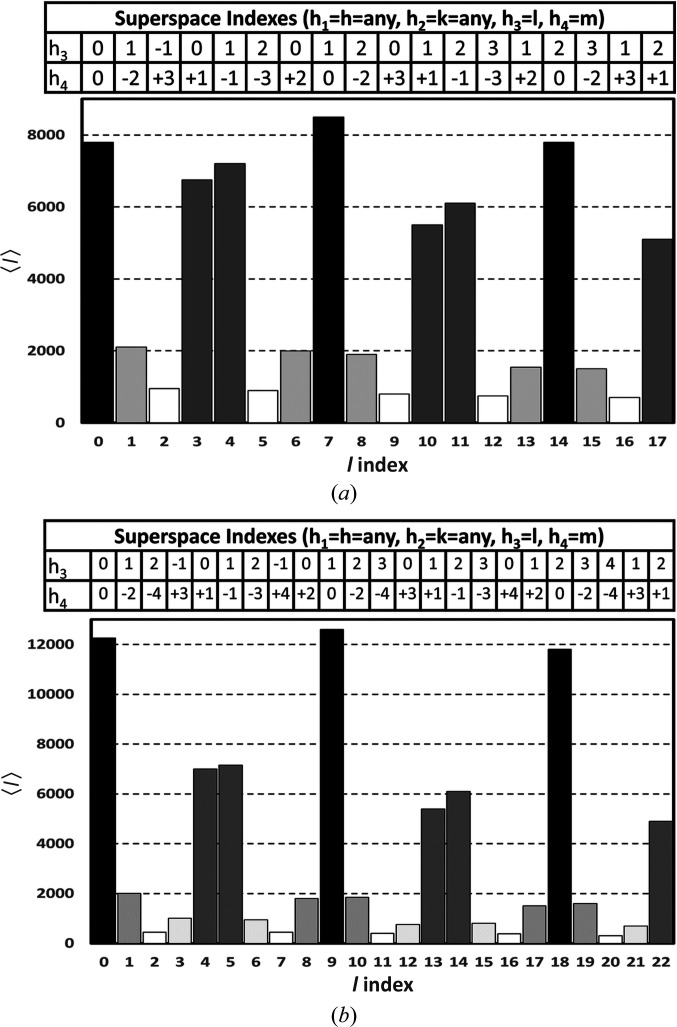
Average reflection intensities as a function of the *l* index. The superspace indexing of the same average reflection intensities (*h*_3_ and *h*_4_) is shown for (*a*) PDB entry 4n3e and (*b*) PDB entry 6sjj. These charts were adapted from Sliwiak *et al.* (2015 ▸) and Smietanska *et al.* (2020 ▸).

**Figure 3:**
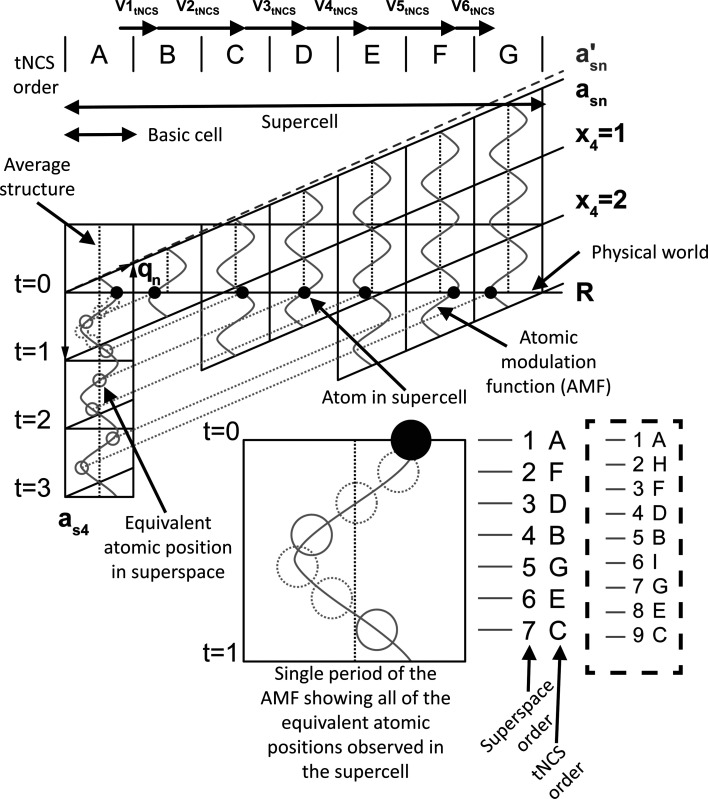
Superspace diagram for (3+1)D superspace with three periods of the modulation function every seven basic cells. A periodic function in superspace is used to describe the seemingly random appearance of tNCS-related atoms in a supercell (along **R**, where **R** represents 3D space in the physical world). These displacements are extremely exaggerated as the modulations are typically small compared with the unit cell. An average structure position is given by the vertical dotted line. The **q**_*n*_ vector determines the angle between **x**_4_ and physical space (**a**_s*n*_). The enlarged region for *t* = 0 to *t* = 1 shows the reordering of basic cells that occurs in superspace. In the dashed box to the right of the 3/7 reordering is the reordering for the 4/9 supercell. The grey 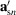 line shows the vector that would need to be used for a 4/9 modulation; although not shown, this line would interect at four periods after nine basic cells, leading to a ninefold supercell.

Superspace, as developed by Janner and Janssen (Janner & Janssen, 1977 ▸, 1980*a* ▸,*b* ▸), describes how periodic atomic functions in higher dimensional space can be used to explain the diffraction patterns of modulated structures observed in physical space. This theory provides a very powerful approach that even allows the indexing of diffraction patterns of incommensurately modulated structures and interpretation of the resulting structures. These types of structures have a very distinct diffraction pattern of main reflections with associated satellites. For diffraction, the superspace approach uses the concept of **q** vectors to describe the location of satellite reflections relative to their main reflection. The number of **q** vectors also indicates the number of extra dimensions needed to describe the modulation. The easiest modulation case is described using (3+1)D superspace, where only one **q** vector is needed to describe the satellite reflections. The **q** vector (or the spacing between the main reflection and the first-order satellite) that describes the diffraction pattern of the commensurately modulated structure observed for the PDB entry 4n3e data can be determined from an analysis of the average reflection intensities arranged as a function of *l* (Sliwiak *et al.*, 2015 ▸; Fig. 2 ▸ *a*). In the same manner, the **q** vector for PDB entry 6sjj (Smietanska *et al.*, 2020 ▸) can be determined by a similar analysis (Fig. 2 ▸ *b*). The intensity relationship for main reflections and satellites is that on average first-order satellites will be less intense than the main reflection, second-order satellites will be less intense than first-order satellites, and so on. Locally, this trend may not hold due to the actual shape of the AMFs. Averaged over many reflections, the trend in reflection intensities will hold. Fig. 2 ▸(*a*) shows that the first-order satellites are spaced three reflections away from each main reflection, and other related satellites and the main reflections are at positions divisible by seven as a function of *l*. Taken together, main reflections every seven reflections and first-order satellites three reflections away from the main reflections lead to a **q**-vector definition of 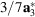. Using the 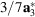 **q** vector, which can be interpreted as having three periods of the periodic modulation function every seven basic cells, allows the construction of a superspace diagram (Fig. 3 ▸) to demonstrate how a periodic AMF in this higher dimensional space (grey wavy periodic lines in Fig. 3 ▸) can account for the observed atomic positions (black circles in Fig. 3 ▸) in the supercell where the AMFs intersect with physical space (**R** in Fig. 3 ▸). Due to the periodicity of the AMF, all of the observed atomic positions in the supercell can be translated to a single period of the AMF in *t* (Fig. 3 ▸, enlarged area), where *t* has a value from 0 to 1 over a single period. In superspace, data are usually shown as a function of *t* (lines in superspace but parallel to **R**) or **x**_4_ (lines parallel to **a**_s*n*_). The choice depends on the information that is trying to be conveyed. Additionally, it is possible to see that the states encountered in the supercell by traversing the basic cells in physical space (A–G) are not in the same order as encountered in superspace (1–7) (enlarged area in Fig. 3 ▸). For (3+1)D superspace, the AMFs are line functions, where the **x**_4_ value of the position is determined by the dot product of the average position of the atom in the basic cell in fractional coordinates with the **q** vector plus a phase offset *t*,
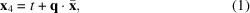
where
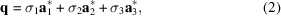


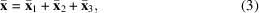


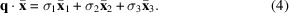


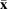
 is the average position of an atom in a basic cell in the crystal and σ_*n*_ is the **q**-vector coefficient. All atomic parameters in superspace are governed by AMFs.

In the case of the reflection data for PDB entry 6sjj, the main reflections (highest intensity) appear for values of *l* that are divisible by 9. The next highest (first-order satellites) appear four reflections away. This leads to a **q**-vector of 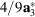. In this case, there are four periods of the modulation wave for every nine basic cells. The reordering of the basic cells in superspace for this 4/9 case is shown in the dashed box in Fig. 3 ▸.

## 2. Methods   

**Figure 4:**
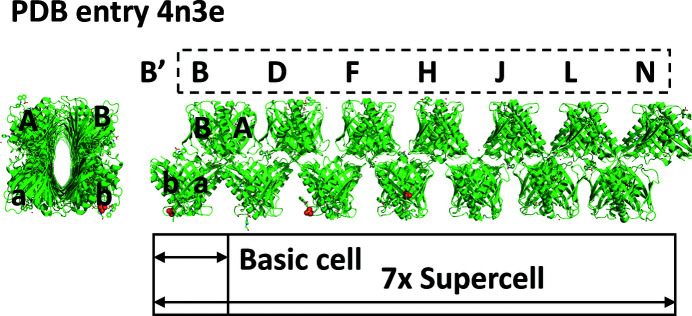
The superstructure of PDB entry 4n3e with views looking down and along the structure. The supercell is a sevenfold expansion in the **c** direction. Chains in the first basic cell (a, b, A and B) use a notation with a prime to denote all related chains in subsequent basic cells; for example, B′ refers to chains B, D, F, H, J, L and N. The PDB entry 6sjj supercell looks similar but has two additional basic cells and uses a slightly different naming scheme for the chains.

Details of the processing will focus on the PDB entry 4n3e structure as an example. The processing is the same for the PDB entry 6sjj structure, with the primary difference being that the supercell is a ninefold expansion and the **q** vector is 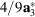 as opposed to 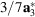. The PDB entry 4n3e structure consists of a sevenfold basic cell expansion into a supercell with four protein chains and some small molecules in the asymmetric unit (ASU) of the basic cell where the modulation was along the **x**_3_ dimension (Fig. 4 ▸). The resulting supercell has 28 chains in the ASU. The chains composing the first basic cell are A, B, a and b. All of the chains related by the modulation in other individual basic cells will be annotated with a prime. For example, B′ represents the chains in basic cells 1–7 (B, D, F, H, J, L and N), which are all related by tNCS to the B chain in the first basic cell (Fig. 4 ▸). The PDB entry 6sjj structure consists of a ninefold basic cell expansion into a supercell (not shown), and like the PDB entry 4n3e structure has four protein chains in the ASU of the basic cell. In this case, chains j′, B′, i′ and A′ of PDB entry 6sjj correspond to chains b′, B′, a′ and A′ of PDB entry 4n3e. Superspace figures for chain B′ of PDB entry 4n3e have previously been published (Lovelace & Borgstahl, 2020 ▸) but the processing details of how they were generated were not discussed.

**Figure 5:**
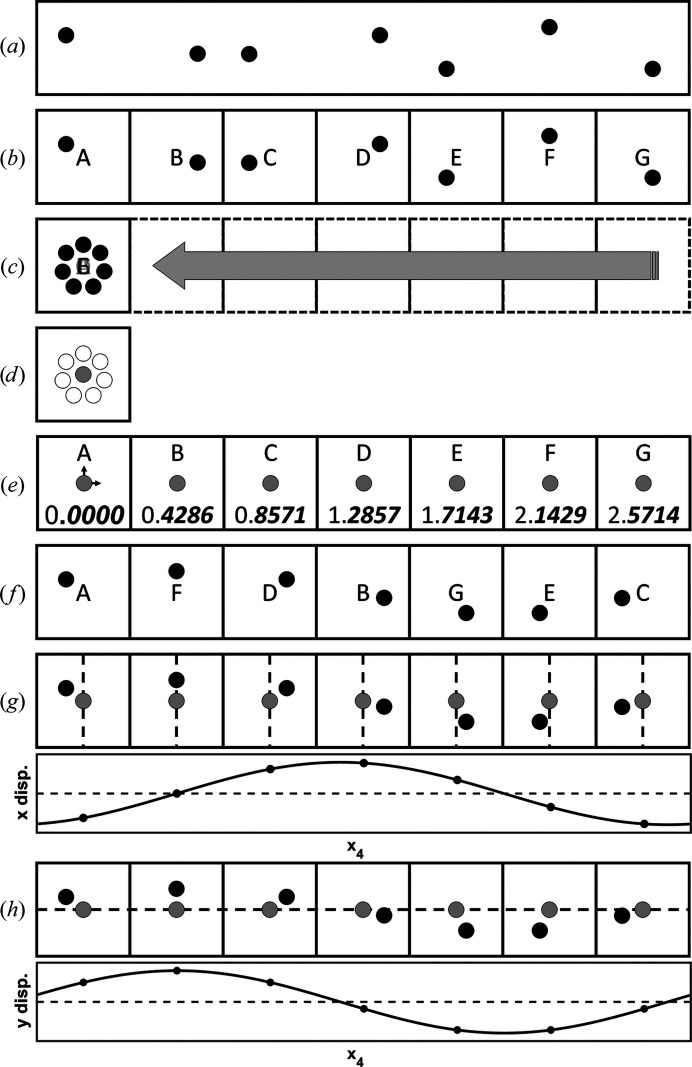
The process of converting a supercell into its subspace representation: (*a*) the supercell, (*b*) partitioning of the supercell into the basic cell, (*c*) translation of all of the basic cells to overlap with the first basic cell, (*d*) calculation of the average positions from the translated positions of related elements, (*e*) using the average position in each basic cell to determine **x**_4_, (*f*) sorting the basic cells by fractional **x**_4_, (*g*) determining horizontal displacements from the average as a function along **x**_4_ and (*h*) determining vertical displacements from the average as a function of **x**_4_.

*Matlab* (MathWorks) scripts were used to manipulate the PDB files to display the data in superspace. These scripts are available in the supporting information and will generate many of the figures shown in this paper. The process of breaking down a supercell (Fig. 5 ▸ *a*) and transforming it into superspace starts with dividing it into basic cells (Fig. 5 ▸ *b*). The number of basic cells is the product of the denominators of the rationalized **q** vector. The number of basic cells along any dimension will be equal to the denominator of the **q** vector in that dimension. For PDB entry 4n3e the number of total basic cells is seven because 1 × 1 × 7 = 7 and they are along **x**_3_ because that is the only dimension with a denominator (σ_3_ = 3/7) greater than 1. The function *superorder.m* (provided in the supporting information) will return a rationalized **q** vector to within a tolerance given the **q** vector in decimal form. The basic cells are then all translated to the coordinates of a single basic cell (Fig. 5 ▸ *c*). An average atomic position (grey dot in Fig. 5 ▸ *d*) is calculated averaging the related basic cell atoms (grey circles in Fig. 5 ▸ *d*). The average position is used to calculate an **x**_4_ position for that atom in all of the basic cells (Fig. 5 ▸ *e*). Sorting can then be performed on the fractional component of **x**_4_ to determine the frame order to animate the basic cell (Fig. 5 ▸ *f*). Additionally, plots of the approximated AMFs can be created by measuring the displacement from the average position of an atom (black circle) for a single dimension (dashed line) (**x**_1_ in Fig. 5 ▸ *g* or **x**_2_ in Fig. 5 ▸ *h*) and then plotting this information as a function of **x**_4_.

**Figure 6:**
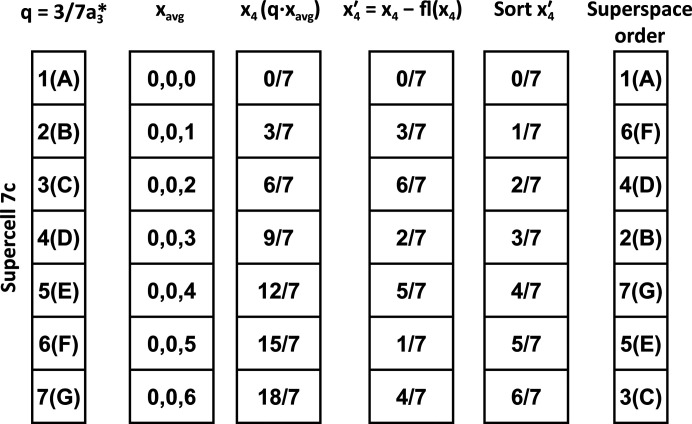
Sample calculations were performed by the *superorder.m Matlab* script, where the basic cells are assigned a numerical index (1, 2, …, *n*) and an average position that is the same in each basic cell (**x**_avg_). The value of **x**_4_ is calculated by the dot product of **q** with **x**_avg_. The **x**_4_ values are translated to the range 0 to 1, resulting in 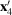, using the floor function, which returns the lowest integer value. 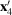 is sorted and the resulting reordered indices are returned.

The values in **x**_4_ were calculated (Fig. 6 ▸) using the *Matlab* function *superorder.m* (available in the supporting information). Firstly, *superorder.m* assigns a numerical index to the basic cells and then uses the most straightforward starting position (0, 0, 0) and determines equivalent positions in all of the basic cells. Next, it calculates **x**_4_ and then extracts the fractional part 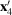 by subtracting the lowest integer from the value using the floor function. The floor function is used so that negative values are correctly calculated. The basic cells are sorted by 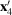 and the basic cell sort order is returned as a list of the numerical indices as well as the associated values of 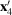.

The *superorder.m* script will work with full 3D supercells for (3+1)D superspace (a single **q** vector with all nonzero components). The supporting information provides a 2D supercell (along **x**_1_ and **x**_3_) example found in Wagner & Schönleber (2009 ▸). In that paper there are two plots, one of the supercell (Fig. 15) and one showing the basic cells of the supercell rearranged in superspace order (Fig. 23). Although they do not discuss how they performed this rearrangement, the *superorder.m* function can determine the necessary reordering of the 35 basic cells and reproduce the published results.

## 3. Results   

**Figure 7:**
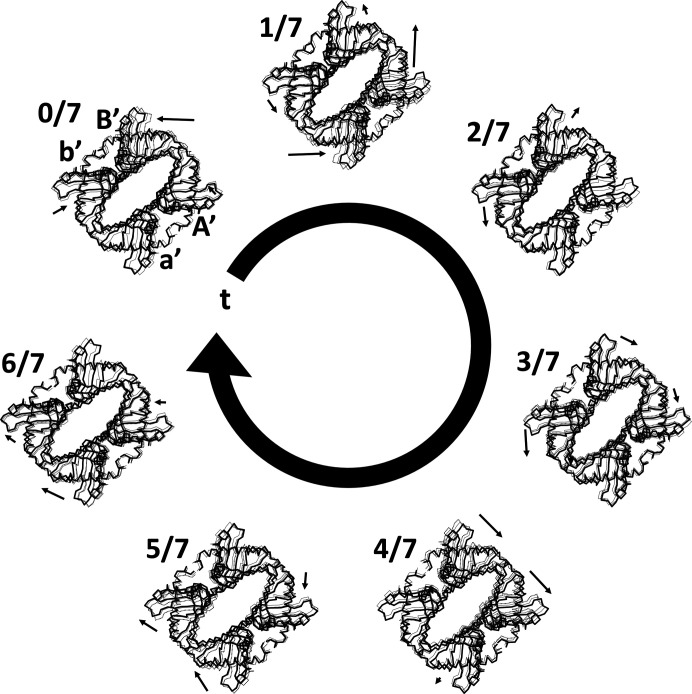
PDB entry 4n3e basic cells arranged in periodic superspace order (*t* = 0 to *t* = 1). Asymmetric unit basic cells were plotted to show the current data (black line; C^α^ trace of the four chains a′, A′, b′ and B′) as well as the previous basic cell (black dashed lines) and average position (grey line). The arrow indicates the relative displacement from the previous basic cell. Arrows are on the same relative scale for all basic cells.

The results of transforming the supercell of PDB entry 4n3e into superspace (Fig. 7 ▸) demonstrates the power of this method to make the underlying periodic modulation accessible for analysis. Fig. 7 ▸ shows the basic cell states in a circle arranged by the value of *t*. The value of *t* was calculated using the corner of the basic cell as the origin. It is shown as a circle to reinforce the idea that AMFs are periodic along the **a**_s4_ direction. The rearrangement of the basic cells transforms what previously appeared to be random motions between neighbouring basic cells in the supercell into a cleaner sequence of consecutive transitions. It is more visually appealing and easier to visualize this by watching the movie (supporting information file 4n3e_ans-3-7-mas.gif), which shows the basic cells as frames animated in superspace order. These seven snapshots of the continuous AMFs in (3+1)D superspace show that most of the modulation (as viewed down **x**_3_) is related to an opening and closing of the space between chains b′ and B′ (Fig. 7 ▸). The modulation is not smooth in terms of the amount of displacement between frames being similar. Some consecutive frames show a large amount of displacement relative to the previous frame and other frames show very little relative displacement. AMFs that were not smooth were expected because of the higher order observed satellites, which are indicative of more sharp displacements. Additionally, all of the chains modulate relative to the average structure. Black arrows show the relative displacement of the four chains in each basic cell relative to the previous position. The black dashed line is the previous state and the grey line is the average position. When the same analysis is applied to PDB entry 6sjj it shows a very similar modulation (supporting information file 6sjj-ans-4-9-mas.gif). With an understanding of how to transform the data into superspace, an interesting question would be: is there a relationship between the two 3D superstructures in (3+1)D superspace?

**Figure 8:**
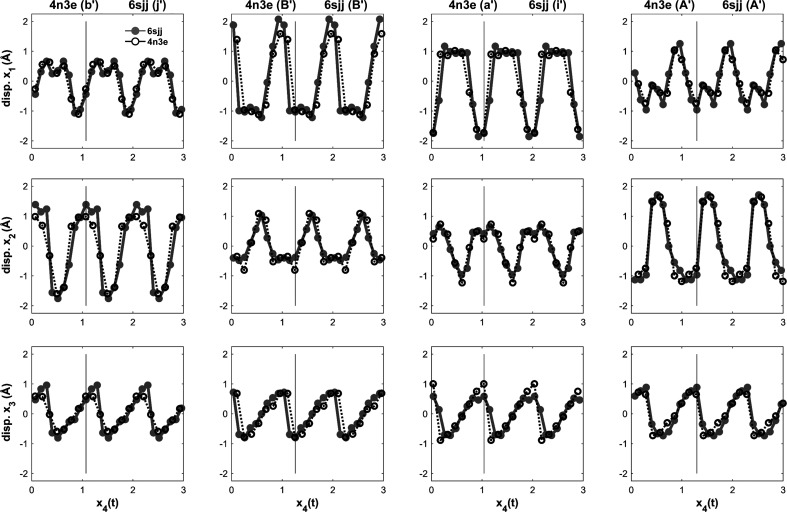
AMF approximations using the average centre of mass for each of the four chains in the ASUs for PDB entry 4n3e (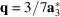) and PDB entry 6sjj (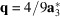) in the **x**_1_, **x**_2_ and **x**_3_ directions as a function of **x**_4_.

PDB entries 4n3e and 6sjj were compared by generating AMF approximations for the centre-of-mass displacements with respect to the three crystal directions **x**_1_, **x**_2_ and **x**_3_ of the four chains in the basic cell ASU. Fig. 8 ▸ shows plots of the displacements (in each of the three primary axes: **x**_1_, **x**_2_, **x**_3_) as a function of **x**_4_ (Fig. 8 ▸) for both PDB entry 4n3e (black circles and dashed black lines) and PDB entry 6sjj (grey dots and grey lines). Data from both superstructures overlay with each other in (3+1)D superspace. These superspace-plot data support the idea that both observed superstructures are 3D intersections in physical space from the same (3+1)D structure. In their paper, Sliwiak and coworkers observed that the change between the two structures was quite large given that the only difference in the crystallization conditions was the addition of melatonin (Smietanska *et al.*, 2020 ▸). They believed that this addition would result in at most a small change to the structure. They had also hoped to see melatonin binding to the complex. Unfortunately, they did not observe melatonin binding in the electron-density maps. In (3+1)D superspace, melatonin does appear to make a small change to the **q** vector (less than 1° rotation) and this small change results in changes to the intersection of the higher dimensional structure with physical space, resulting in the observed increase in supercell dimensions as well as the changes to the arrangement of the chains within the supercell compared with the PDB entry 4n3e structure.

Investigation of the AMFs reveals some interesting properties that apply to both the PDB entry 4n3e and 6sjj structures. The AMFs for **x**_1_ and **x**_2_ seem to be mirrored representations, relative to **x**_4_, between b′/j′ and A′ as well as between B′ and a′/i′. In **x**_3_, all four chains follow a sawtooth function. The sawtooth function increases linearly up to some maximum followed by a discontinuous or nearly discontinuous reset back to a minimum, where it then begins the linear increase again. The major difference between the four chains is where the reset of the sawtooth occurs in **x**_4_. Chains a′/i′ and A′ experience the reset simultaneously in the same basic cell (vertical black lines in Fig. 8 ▸), whereas chain b′/j′ is lagging and has not reached the peak of the sawtooth and chain B′ has already experienced the sawtooth reset.

The PDB entry 4n3e structure has another interesting superspace feature where there are some occupancy modulations for some of the bound small molecules. An example of an AMF for a small molecule near **a**′ is shown in the supporting information. Superspace represents occupancy modulations by having no value for the AMF at these positions by using a function with a discontinuity.

Another aspect that can be explored is the (3+1)D space group. In this case we are going from the information provided in the original papers to arrive at the correct (3+1)D space group. Both structures were in space group *C*2. Using *Superspace Group Finder* (https://it.iucr.org/resources/finder/; Orlov *et al.*, 2008 ▸) and inputting *C*2 in the 3D group set results in 21 potential parent (3+1)D space groups. These space groups can be further reduced using the directions provided in Porta *et al.* (2017 ▸) by first limiting the list to those that are possible for protein crystals and then reducing the remaining space groups to those which match the observed parameters of the **q** vector. In this case, there is only one remaining (3+1)D space group: *C*222_1_(00γ). Table 1 ▸ summarizes the space-group data. The main difference between the two (3+1)D unit cells is that the **q** vectors are slightly different (0.015). The superspace group indicates that there should be a screw axis down **a**_3_. The previously observed relationships in the AMFs between b′/j′ and A′ as well as between B′ and a′/i′ seem to support a screw axis as expected for the (3+1)D space group.

## 4. Conclusions   

Looking at modulated atomic structures in superspace has provided an insightful understanding of how two modulated structures that are related in (3+1)D superspace appear very different in physical space. In this case, the difference can be attributed to slightly different 3D intersections of the same (3+1)D structure caused by a small change in the **q** vector. Additionally, these structures provided validation of the approaches that we are using to evaluate our PA refinements. They also provide independent validation of the tNCS refinement approach that was originally used to solve these structures. By providing the associated scripts as supporting information, we hope that other research groups will be able to more easily perform this analysis in the future on other modulated structures.

## Supplementary Material

ZIP file containing all supporting information. DOI: 10.1107/S2059798321003740/rr5202sup1.zip

## Figures and Tables

**Table 1:** Space-group and unit-cell data for PDB entries 4n3e and 6sjj

PDB code	4n3e	4n3e, (3+1)D	6sjj	6sjj, (3+1)D
Space group	*C*2	*C*222_1_(00γ)	*C*2	*C*222_1_(00γ)
*a*, *b*, *c* (Å)	146.29, 146.29, 298.56	146.29, 146.29, 42.65	145.85, 145.85, 385.4	145.85, 145.85, 42.83
α, β, γ (°)	90, 90, 90	90, 90, 90	90, 90, 90	90, 90, 90
**q**_1_		0, 0, 3/7		0, 0, 4/9
