# Supplementary material for: Are the St John’s wort Hyp-1 superstructures different?
Source: Acta Crystallogr D Struct Biol. 2021 May 14;77(Pt 6):790–8. doi: 10.1107/S2059798321003740 (PMC8171068; doi:10.1107/S2059798321003740)
Supplement: Supplementary file 1 [file d-77-00790-sup1.zip › review_202001.pdf]

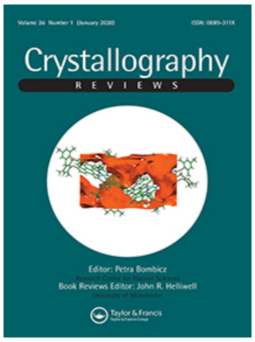

Taylor & Francis
Taylor & Francis Group

Crystallography Reviews

ISSN: 0889-311X (Print) 1476-3508 (Online) Journal homepage: https://www.tandfonline.com/loi/gcry20

Characterizing pathological imperfections in macromolecular crystals: lattice disorders and modulations

Jeffrey J. Lovelace & Gloria E. O. Borgstahl

To cite this article: Jeffrey J. Lovelace & Gloria E. O. Borgstahl (2020) Characterizing pathological imperfections in macromolecular crystals: lattice disorders and modulations, Crystallography Reviews, 26:1, 3-50, DOI: 10.1080/0889311X.2019.1692341

To link to this article: https://doi.org/10.1080/0889311X.2019.1692341

Published online: 10 Dec 2019.

Submit your article to this journal

Article views: 43

View related articles

View Crossmark data

Full Terms & Conditions of access and use can be found at
https://www.tandfonline.com/action/journalInformation?journalCode=gcry20

CRYSTALLOGRAPHY REVIEWS
2020, VOL. 26, NO. 1, 3–50
https://doi.org/10.1080/0889311X.2019.1692341

Taylor & Francis
Taylor & Francis Group

Check for updates

REVIEW

Characterizing pathological imperfections in macromolecular crystals: lattice disorders and modulations

Jeffrey J. Lovelace and Gloria E. O. Borgstahl

The Eppley Institute for Research in Cancer and Allied Diseases, University of Nebraska Medical Center, Omaha, NE, USA

ABSTRACT
Macromolecular crystal structure determination can be complicated or brought to a halt by crystal imperfections. These issues motivated us to write up what we affectionately call 'The Definitive Hitchhiker's Guide to Pathological Macromolecular Crystals: Lattice Disorders and Modulations'. Perhaps the most challenging imperfections are lattice order–disorder phenomena and positional modulations. Many of these types of crystals have been solved, and progress has been made on the more challenging forms. Diagnostic tools and how to solve many of these problem crystal structures are reviewed. New avenues are provided for approaching the solution of incommensurately modulated crystals. There are a good number of case studies in the literature of lattice order–disorder phenomena and crystallographic modulations that make it timely to write a review. This review concludes with a projected pathway for solving incommensurately modulated crystals, personal views of future directions and needs of the crystallographic community.

ARTICLE HISTORY
Received 6 May 2019
Accepted 10 November 2019

KEYWORDS
Lattice-translocation defects; rotational order–disorder; superlattice; commensurate; incommensurate; translational noncrystallographic symmetry

Contents

PAGE

1. Introduction 5
   1.1. Historical example 6
   1.2. A brief history of pathological macromolecular crystals 7
2. Software programmes 10
   2.1. Twin analysis 11
      2.1.1. XPREP 11
      2.1.2. TRUNCATE 11
      2.1.3. UCLA Twin Detection server 11
      2.1.4. Phenix.xtriage 11
   2.2. Indexing and integration 11
      2.2.1. Twinsolve 11
      2.2.2. Eval15 11
      2.2.3. DIALS 12
   2.3. Structure solution and refinement 12

CONTACT Gloria E. O. Borgstahl gborgstahl@unmc.edu

© 2019 Informa UK Limited, trading as Taylor & Francis Group

2.3.1. MOLREP 12
2.3.2. Phaser 12
2.3.3. JANA2006 12
2.3.4. REFMAC5 13
2.3.5. Phenix.refine 13
2.3.6. MAIN 13
2.3.7. Superflip 13
2.4. Data visualization 13
2.4.1. Coot 13
2.4.2. Pymol 14
2.4.3. Matlab 14
2.4.4. RStudio 14
3. Rotational order – disorder (ROD) 14
3.1. What is it? 14
3.2. Symptoms 14
3.3. Solution 15
3.4. Case studies 15
3.4.1. rsTagRFP 15
3.4.2. Stefin B 18
4. Lattice-translocation defects (LTD) 21
4.1. What is it? 21
4.2. Symptoms 21
4.3. Solution 21
4.4. Case studies 22
4.4.1. MVV $IN_{NTD+CCD}$–$LEDGF_{IBD}$ complex 22
4.4.2. CRMP-4 25
5. Translational non-crystallographic symmetry (tNCS) 26
5.1. What is it? 26
5.2. Symptoms 27
5.3. Solution 27
5.4. Case studies 28
5.4.1. Hyp-1-ANS 28
5.4.2. FBPase 29
6. Incommensurately modulated crystals 32
6.1. What is it? 32
6.2. Symptoms 33
6.3. Solution 33
6.4. Case studies 35
6.4.1. Profilin:actin (PA) complex 35
6.4.2. Hyp-1-ANS complex 37
6.4.3. N-acetyl-neuraminic lyase (NAL) 41
7. Conclusions 41
Acknowledgments 43
Disclosure statement 43

Funding 43
Notes on contributors 44
ORCID 44
References 44
Index 48

1. Introduction

Screening crystals for a good quality diffraction pattern can be challenging. Proteins themselves are flexible by nature, and additionally, protein crystals contain a large amount of solvent. Together these properties make it easier for protein crystals to incorporate impurities leading to lattice imperfections during crystal growth. Often sub-optimal crystals can be discarded at the crystal screening stages. On occasion, there are crystals that diffract with sharp strong reflections during the initial screening, but it is only after collecting a full dataset and attempting to process it that problems arise.

The most common problems that occur are twinning or multiple crystals that are diffracting at the same time. Twinning [1–3], is defined as a case where distinct three-dimensional domains of a crystal are oriented differently but are otherwise well ordered individually. In many cases, twinning can be recognized and dealt with. There are many examples in the literature and excellent tools to help detect and work with twinning to solve structures and this area has been well-reviewed [1–5] and it continues to be an area where improvements can be made. Recently, multiple crystals diffracting simultaneously has become an issue when using streams of nanocrystals with XFEL sources [6] and software has become available to deal with this type of multiple crystal diffraction [7].

In this review, we focus on pathological crystals that can be loosely grouped into four classes: rotational order–disorder, lattice-translocation defects, translational and rotational non-crystallographic symmetry, and modulated structures. We present the cases in the order of simplest to most difficult to solve. In the worst cases, a crystal may belong to multiple categories and may also be twinned. These four types of pathologies occur in up to 5% of samples [8]. The literature is sparse on these types of examples because many times these crystals and their diffraction data are discarded and remain unsolved and unpublished. Fortunately, there are courageous researchers who refused to give up and persevered in working through these challenges. Sometimes there was just no other option (i.e. this was the only sample that diffracted). In other instances (e.g. incommensurately modulated profilin:actin), crystals were forced into a pathological state to study structural intermediates of a biological process.

How can one tell if they are 'lucky' enough to have encountered one of these samples? The simple answer is – it depends. Usually, there is some indication of an issue when the diffraction pattern is indexed, integrated and scaled. For example, not all of the reflections may be predicted or indexed correctly or the reflection statistics are much worse than expected. It may be useful to examine the raw diffraction images to see if there are regions where the diffraction peaks go from round single reflections to very elongated ellipsoids (order–disorder or lattice-translocation defects) or some reflections are not integrated/indexed (incommensurate modulation). More often the

problems show up later in the structure solution pipeline during phasing and refinement. Sometimes these pathological issues can be mitigated just by adjusting the crystallization process and this has been reviewed elsewhere [9–13]. A partial but by no means complete list of options includes: cutting off disordered protein regions, mutating amino acids in crystal contacts, and adjusting crystallization solutions (concentrations, pH, chemicals). We want to encourage the community to not reject and discard their problem crystals but instead to at least record diffraction images and store them in a repository for future analysis. An example of such a repository can be found at https://researchdata.ands.org.au/diffraction-image-experiment-repository.

1.1. Historical example

It has been suggested that the driving forces that allowed problematic types of crystallographic problems to be recently solved are improved X-ray sources, detectors and cheap, abundant processing power. That view is not entirely accurate because even in the earliest days of macromolecular crystallography these issues were encountered, studied and remarkable insights were derived from the data, impressive considering the lack of detectors, computers, and software at the time. The earliest known example for a pathological protein crystal was from a collection of papers published by Bragg, Howells, Perutz, and Cochran in 1954 [14–16]. They were investigating crystals of horse methemoglobin with and without imidazole. Using a precession camera, they collected diffraction images on X-ray film from each of the principal axes. For the imidazole crystal form, they noticed that the diffraction pattern contained layers of sharp reflections followed by layers of streaky reflections (Figure 1).

Analysis of what was occurring in these crystals was aided by the fact that there was another crystal they had grown that was well behaved (no imidazole). The researchers were able to compare the unit cells and space groups of the two crystals (Figure 2) to formulate what was different between the two crystal forms. The well-behaved sample had a monoclinic unit cell (Figure 2(a)) with $a = 109.2$ Å, $b = 63.2$ Å, and $c = 57.4$ Å and $\beta = 110°$. The imidazole sample had an orthogonal unit (Figure 2(b)) cell of $a = 109$ Å, $b = 63$ Å, and $c = 104$ Å. As it turns out, c*sin(β) = 51.2 Å for the monoclinic cell is about half of the c dimension for the orthorhombic case. From this, the authors concluded that the

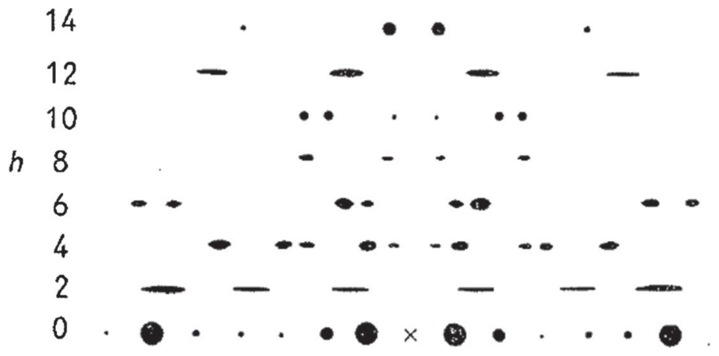

Figure 1. Sketch of the reflections observed for imidazole bound methemoglobin crystals [16]. Reproduced with permission of the International Union of Crystallography.

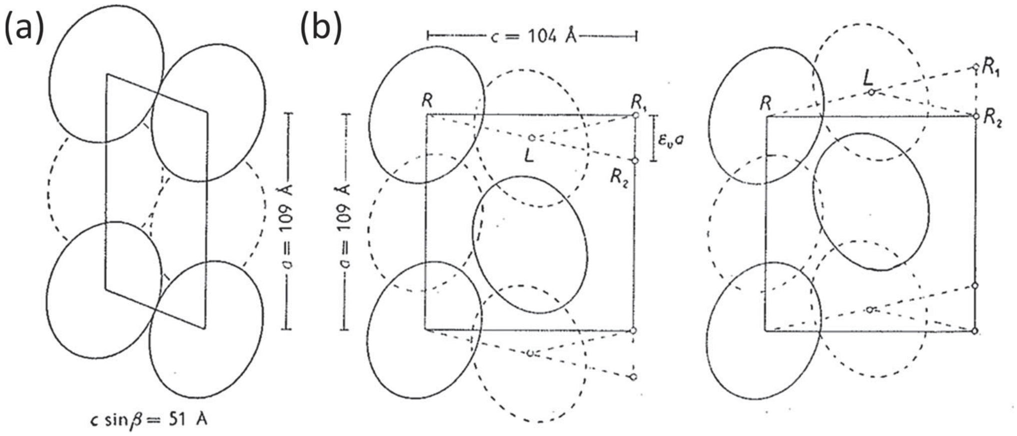

Figure 2. Schematic of the unit cell packing for the normal crystals (a) and the crystals with LTD defect (b) showing two possible positions of the lattice translocation [16]. Reproduced with permission of the International Union of Crystallography.

arrangement of planes within the crystal was similar between the two molecules, but in the case of the orthorhombic cell, every other plane had the molecules tilted in the opposite direction. These tilted planes can then bind in one of two possible orientations (limited by the c*sin(β) as in Figure 2(b)). The probability of the plane shifting to one of the two options is random within the crystal which lead to the observed smeared out reflections (Figure 1). Eventually, they classified this case as a layered structure containing random displacements. Today, this crystal would fall under the umbrella of a lattice-translocation defect (LTD).

1.2. *A brief history of pathological macromolecular crystals*

Disclaimer: The focus of this review is centred on macromolecules and from time to time includes developments in small molecule crystallography that are mostly related to how the history of how the theory developed. The reason for including some small molecule work here was to mainly demonstrate that there tends to be a lag between when the phenomenon was identified in small molecule crystallography and when a sample showing the same phenomenon was found in macromolecular crystallography. This does not mean that all work on these types of issues in small molecules stopped being important or that publications haven't been occurring in those areas, they have, it is just that those items are not a focus of this review. Details of theory and implementations are provided in the related example sections because the mathematical solutions are problem-dependent.

In the world of small molecules, pathological crystals were observed a few decades earlier and researchers were coming up with the math to describe these observations. Around the same time (in the mid to late 1950s) that Howells and co-workers were reporting their results, mathematical theories were being proposed for the more generalized case of the phenomenon known as Order–Disorder [17,18] based on results observed in small molecules. These types of defects are best described as the incorporation of ordered regions into the crystal in a disordered or partially disordered way. LTD would be a subset of this type of crystal.

On the protein front in the 1960s, things remained quiet in terms of reported pathological crystals with the lone stand out example reported by Glauser and Rossmann [19] about erythrocyte catalase crystals which appeared to have LTD, but they decided they could process it no further. During the 1970s theoretical models were proposed to solve a perplexing issue observed in small molecule crystals where sharp Bragg reflections were observed in diffraction data but they could not all be indexed using a single unit cell and the samples where not twinned or suffering from some other known issue. Researchers were convinced the extra satellite reflections (the reflections that could not be indexed and generally less intense then the reflections that were indexed) could be described by a periodic displacement from the average position overlaying itself on the unit cell but with a period that was incommensurate with the unit cell. The difficulty was how to incorporate this model into the crystallographic theoretical framework. The solution that was arrived at to deal with these incommensurately modulated samples was to use higher-dimensional space. Through a series of papers Janner, Janssen and de Wolff laid out the theory and how it could be applied [20–24]. Much like the framework used to describe Order–Disorder, this higher-dimensional solution was of limited use for many years until computing resources, and nifty mathematical tricks to simplify the application of the math in digital computers. One of the most straight famous examples would be the Fourier Transform [25] which was discovered in the 1800s. This led to the discrete Fourier transform (with a computational of complexity N^2) [26]. This was reformulated as the Fast Fourier Transform (FFT) [27] (with a complexity NLog(N)). As of today there is freely available as library of optimized FFTs distributed as the Fastest Fourier Transform in the West Library (FFTW) [28] (some implementations achieve a computational complexity of $^N/_2Log_2(N)$). The improvements in computation complexity have made it possible to run the transform in real-time and it has had major impacts across all area of digital signal processing including crystallography.

Towards the end of the 1980s and beginning of the 1990s, a couple of new crystals were reported. Pickersgill [29] published diffraction images for crystals of spinach ribulose bisphosphate carboxylase showing sharp reflections interspersed with elongated reflections, a classic feature of a crystal with LTD. Due to the limitations of computing power they were unable to process the data further. In another paper reported by Brik and coworkers about the structure of tyrosyl-tRNA synthetase [30] disordered regions are mentioned and something reminiscent of an order–disorder arrangement, but they do not show any diffraction data, so it is difficult to determine if this is some form of LTD. Schutt and colleagues published the diffraction pattern of an incommensurately modulated protein crystal of profilin:actin complex (PA) in 1989 but did not recognize it as such and called it the 'semicrystalline state' [31]. They did go on to correctly guess that they saw some sort of modulation in the atomic positions associated with a conformational change in actin. The pattern was incommensurately modulated so the diffraction data could not be indexed and no further work was attempted on this structure for nearly 20 years. During the 1990s work on pathological crystals was directed towards twinned crystals and how to process these data [1–4,32–34].

Even though the primary focus was on twinning as far as the problematic crystal cases went in the 1990s, now and then a different type of pathological crystal would be reported. For example, Merritt and coworkers [35] discussed a diffraction pattern that could have been from a modulated sample, but they only briefly mention the issue with no discussion or publication of diffraction images showing the problem. From the description of the

diffraction pattern and their mention of the idea of using a superlattice, it could be that this sample was a modulated structure (possibly incommensurate as presumably, the superlattice approach failed to index the sharp reflections) as opposed to a sample with LTD which would have contained the streaky reflections interspersed with sharp reflections.

In the 2000s there is an uptick in the number of reported pathological crystals. Importantly, during this time frame, some methods were developed and tested that allow some of these samples to be solved. One of the first approaches demonstrates how to process LTDs. In this, Kamtekar and collaborators devised a scheme to correct the reflection intensities to remove the LTD from the data [36]. The crystals of protein-primed DNA polymerase of bacteriophage φ29 had the classic symptoms of LTD where some reflections were sharp while others were streaky. The authors noticed that by looking over the Patterson maps that peaks occurred related to the LTD. The approach they took to optimize the amount of correction applied to the reflection intensities was achieved by monitoring the Patterson map [37] to minimizing the LTD peaks. Through a sequence of papers, the method is refined and used on different structures [38–44]. The details of this work will be covered as a case study in the LTD section. These papers demonstrate the power of two methods (average reflection intensity plots and Patterson maps) to help diagnose and characterize LTD other than just observing elongated diffuse reflections interspersed with strong Bragg reflections in diffraction images. The first is an average reflection intensity plot along the direction where the diffuse reflections are arranged to help highlight the pattern of the modulation of the intensity. The other is the Patterson map and the occurrence of unexpected strong peaks at a spacing that is too short to be translational non-crystallographic symmetry (tNCS).

At the end of the 2000s, another type of order–disorder was discovered when the crystal structure of fluorescent protein FP480 (PDB: 3H1R) in the I422 spacegroup was solved [45]. For FP480 the unit cell is made up of a tiling of tetramers where each unit cell consists of two tetramers. The tetramers are roughly square in dimension and have the unique property that they can be incorporated into the lattice two ways (90-degree rotation relative to each other), and both orientations occur about the same fraction throughout the sample evenly. The diffractions pattern had a typical appearance of an LTD where there are diffuse and sharp reflections visible throughout the diffraction. Since the disorder in this case is due to rotation, this type of sample has been placed into a new subset of order–disorder known as rotational order–disorder (ROD). Two more cases have been published (both of which are highlighted in the ROD section as case studies). One was reversibly photoswitchable red fluorescent protein rsTagRFP [46] which was very similar overall to the FP480 structure. The other example is the structure of stefin B [47] which unlike typical order–disorder structures has fairly sharp reflections. In this case, there is an issue every fifth layer of octamers where three conformations exist. Again this is quite similar although a bit more complicated to the FP480 case. The reflections are most likely sharper because only about 20% of the crystal has ROD.

Incommensurate protein crystallography made some progress in the late 2000s to early 2010. Incommensurately modulated diffraction patterns contain sharp satellite reflections around stronger main reflections. The main reflections can be indexed normally however the satellite reflections are incommensurate with the main lattice and cannot be indexed with the same lattice as the mains. Incommensurate diffraction requires a **q** vector to index the associated satellite reflections, more details on the theory and notation used will

be presented in the Incommensurately Modulated Crystals section. Lovelace and friends determined the superspace group from a few diffraction images from a room temperature incommensurately modulated PA crystal using TwinSolve [48]. The indexing indicated an orthorhombic space group with a single **q** vector required to describe the modulated reflections based on the superspace theory developed by Janner and Janssen [21–23]. A few years later Porta and coworkers were able to collect, index, and integrate a complete cryocooled modulated dataset of PA [49] using Eval15 [50]. They also performed simulations to work out approaches to refine these types of structures [51–53]. Refinement of the PA structure is in progress. During this time several other groups published modulated diffraction data [54–56]. The paper by Compeotto and others [54] shows the diffraction pattern of an incommensurately modulated protein with a **q** vector that is not aligned with one of the primary axes. In the structure determination of the ANS complex of St John's wort PR-10 protein [55], the authors took a different approach to solve this commensurately modulated structure. They approached the problem as having translational non-crystallographic symmetry.

Translational non-crystallographic symmetry (tNCS) is where the asymmetric unit is composed of more than one copy of the protein. These multiple copies cause variations in the reflection intensities. They also cause characteristic peaks in the native Patterson map. Where this becomes an issue is during molecular replacement. It can sometimes be challenging to place molecules in these types of structures. Phaser [57] has been at the forefront in dealing with tNCS cases. Over the years, improvements have been added to Phaser to increase the chance that it will handle tNCS automatically. One of the most challenging molecular replacements solved by Phaser was the structure of the ANS complex of St John's wort PR-10 protein [55]. This structure was particularly challenging because there were 28 protein molecules in the asymmetric unit cell. Phaser calculates translation vectors to project the tNCS molecules from one position to the next. By slightly varying these translation vectors it is possible to get a better estimate of the observed modulation in reflection intensity which improves that the chances of finding an acceptable molecular replacement solution. From the Patterson map, it is possible to get a general value for the translation vector. tNCS processing has been very successful in improving the success of molecular replacement within Phaser [55–56,58–63]. Commensurately modulated diffraction cases are a challenging form of tNCS. In these cases the reflections are indexed with a supercell (larger unit cell). Alternatively they can be processed as a modulated sample with a **q** vector. Currently, it is best to use the tNCS description or an approximate of tNCS (in the case of incommensurate modulation) because the software tools are well developed for molecular replacement and refinement but cannot currently handle higher dimensional descriptions.

2. Software programmes

Disclaimer: The following software has been used by the authors or has been used in publications reporting pathological crystals. Descriptions may be biased and are based on our opinions after personally using the software. The description may also include information the software authors have provided on their websites. In our experience, the crystallography software development community is extremely friendly and can provide a great deal of help with these problems. We acknowledge that this is probably an incomplete list.

2.1. Twin analysis

2.1.1. XPREP

Although useful for other tasks with respect to this review XPREP [64] can be used to determine if a dataset is twinned. XPREP is part of the Shelx suite [65].

Status: Actively being developed
Website: http://shelx.uni-ac.gwdg.de/

2.1.2. TRUNCATE

TRUNCATE [66] is part of the CCP4 suite [67] and provides both the Perfect Twinning and Partial Twinning tests for reflection files.

Status: Actively being developed
Website: https://www.ccp4.ac.uk/ (part of CCP4)

2.1.3. UCLA Twin Detection server

The UCLA Twin Detection Server [68] provides an online calculation based on local intensity differences which helps it to be more robust in detecting twinning compared to other approaches that may have difficulty with anisotropic diffraction or pseudo-centering.

Status: Actively being developed
Website: https://services.mbi.ucla.edu/Twinning/

2.1.4. Phenix.xtriage

Phenix.xtriage [69] provides twinning analysis on a dataset as well as a number of other sanity checks. It is distributed as part of the Phenix suite [70].

Status: Actively being developed
Website: http://www.phenix-online.org/

2.2. Indexing and integration

2.2.1. Twinsolve

Twinsolve [48] was included as a module in Rigaku Corporation's Crystal Clear as well available from the author. It has a little known mode that allows it to index and integrate modulated diffraction data. Although it was able to index a modulated protein diffraction pattern [71], the assumptions that it made about reflection shape during integration were not valid for the macromolecular case.

Status: Not being actively developed or supported

2.2.2. Eval15

Eval15 [50] is a powerful predicted-profile fitting method for single crystal X-ray diffraction data on area detectors. It handles all types of crystals from small molecules to proteins. The predicted profiles are based on knowledge about the exact experimental setup. This

programme can index and integrate the toughest crystallographic problems. It is very powerful but, in our experience, has steep learning curve, when compared to some of the other programmes, but Eval15 has been able to index and integrate data that other programmes cannot. For example, it has successfully indexed and integrated multiple incommensurately modulated protein datasets [49,54].

Status: Actively being developed
Website: http://www.crystal.chem.uu.nl/distr/eval/

2.2.3. DIALS

The DIALS project [72] is a collaborative endeavour to develop new diffraction integration software to meet the data analysis requirements presented by recent advances. When completed the DIALS software is supposed to have full support for indexing and integration of modulated proteins and the authors of this review have been asked to provide incommensurate datasets for this purpose.

Status: Actively being developed
Website: https://dials.github.io/

2.3. Structure solution and refinement

2.3.1. MOLREP

MOLREP [73] is a molecular replacement programme in the CCP4 suite [67] of programmes. It offers a wide range of options and functions to perform automated molecular replacement.

Status: Actively being developed
Website: https://www.ccp4.ac.uk/ (part of CCP4)

2.3.2. Phaser

Phaser [57] is a programme for phasing macromolecular crystal structures by both molecular replacement and experimental phasing methods. It incorporates options that allow it to handle structures with a large number of molecules where tNCS is an issue. Phaser is distributed as part of CCP4 [67] and Phenix [70].

Status: Actively being developed
Website: http://www.phaser.cimr.cam.ac.uk/

2.3.3. JANA2006

Jana2006 [74] is a crystallographic programme focused on the solution, refinement, and interpretation of aperiodic crystals including modulated structures. It provides a complete set of tools to process and analyze small molecule modulated samples (powder, crystals, magnetic).

Status: Actively being developed
Website: https://jana.fzu.cz/

2.3.4. REFMAC5

REFMAC5 [75] is a flexible and highly optimized refinement package that is ideally suited for refinement across the entire resolution spectrum encountered in macromolecular crystallography. REFMAC5 is distributed as part of the CCP4 suite [67]. The jelly body refinement options have been extremely valuable to get approximations to modulated protein structures to refine correctly.

Status: Actively being developed
Website: https://www.ccp4.ac.uk/ (part of CCP4)

2.3.5. Phenix.refine

Phenix.refine [76] is a general purpose refinement tool for macromolecular structures. It provides a comprehensive set of options as well as a wide range of user tuneable settings. There is a strong user community as well as excellent online documentation. It is distributed as part of the Phenix suite [70].

Status: Actively being developed
Website: http://www.phenix-online.org/

2.3.6. MAIN

MAIN [77] is a programme for molecular structure determination and validation. One of its strengths is how it can support large structures and those with many subunits.

Status: Actively being developed
Website: https://www-bmb.ijs.si/

2.3.7. Superflip

Superflip [78] solves the phase problem by performing charge flipping in an arbitrary number of dimensions. It has been very successful at solving incommensurately modulated small molecules. Unfortunately, protein diffraction data is not typically of high enough quality to use charge flipping to solve the phase problem.

Status: Actively being developed
Website: http://superflip.fzu.cz/

2.4. Data visualization

2.4.1. Coot

Coot [79] although listed under visualization it is much more. Coot displays maps and models and allows model manipulations such as idealization, real-space refinement, manual rotation/translation, rigid-body fitting, ligand search, solvation, mutations, rotamers, Ramachandran plots, skeletonization, non-crystallographic symmetry and more.

Status: Actively being developed
Website: https://www2.mrc-lmb.cam.ac.uk/Personal/pemsley/coot/

2.4.2. Pymol

PyMOL [80] is a molecular visualization system. Many published images of 3D protein structures were created using this programme.

Status: Actively being developed
Website: https://pymol.org/

2.4.3. Matlab

Matlab [81] is a commercial high-level platform for manipulating and displaying data. Matlab combines a desktop environment tuned for iterative analysis and design processes with a programming language that expresses matrix and array mathematics directly. The programme is very useful for quickly testing out ideas prior to the more labour intensive task of coding those solutions.

Status: Actively being developed
Website: https://www.mathworks.com

2.4.4. RStudio

RStudio [82] provides an interactive environment based on R [83]. It is based on python and provides a general platform for processing and displaying data. There are many libraries available to simplify processing of data in a variety of fields. For example, we used RStudio to test out a superspace restrained refinement approach [84] in combination with REFMAC5 [75].

Status: Actively being developed
Website: https://www.rstudio.com

3. Rotational order – disorder (ROD)

3.1. What is it?

Rotational Order–Disorder (ROD) occurs when a component of the crystallographic asymmetric unit can be incorporated into the lattice with a rotation. It can be viewed as a subgroup of order–disorder. In this case, the rotation does not affect the unit cell dimensions and has equally favourable crystal contacts with neighbouring components as compared to preferred incorporation. Figure 3 shows a schematic of how this type of order–disorder can be incorporated into a crystal. This crystal is composed of tetramers where the unit cell contains two of these tetramers (Figure 3(a)). In Figure 3(b) two of the tetramers have been incorporated into the lattice with a 90-degree rotation.

3.2. Symptoms

The symptoms with this type of disorder include any of the following. Diffraction reflections that appear weak and elongated between strong diffraction reflections. In the solved structure, there is a large amount of overlapping density that is not explained by the model but has features that appear similar but rotated or mirrored relative to the fit model. Many of

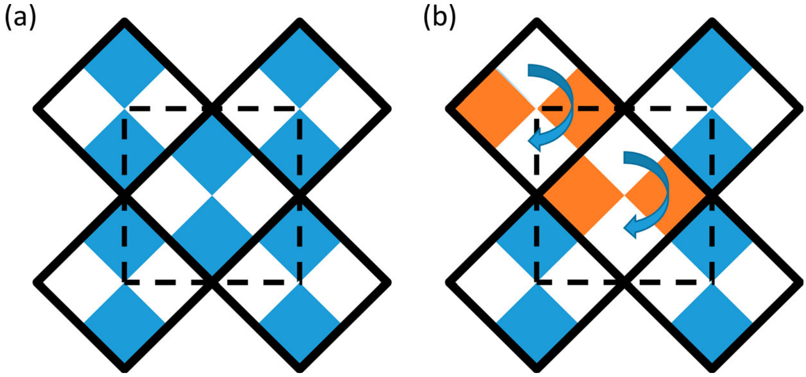

Figure 3. ROD schematic where the solid square lines with four coloured squares inside of them represent a heterotetramer made of two different monomers and the dashed line represents the unit cell of the crystal. (a) the ideal crystal lattice (b) rotational order–disorder incorporation into the lattice.

the reported examples have a potential space group identified by the software as I422 and are composed of tetramers. The Matthew's Coefficient (V_m) calculation [85–87], which uses the unit-cell parameters and molecular weight to predict the multimerization state of the asymmetric unit, gives an unreasonable result. Molecular replacement can have issues finding a solution due to packing function failures. Tests for twinning are negative.

3.3. Solution

Ideally, it would be best to collect the data as if the crystal were in the P1 spacegroup. If this not possible then processing the data as P1 may provide some useful insight even though completeness will be an issue. During indexing and integration the diffuse reflections should be ignored and then the spacegroup should be analyzed for twins. This can be done using available software (TRUNCATE [66], XPREP [64], UCLA Twin Detection Server [68], Phenix.xtriage [69]). The electron density maps should be checked to see if it appears as though there are overlapping electron densities. When overlapping density is observed, an overlapped rotated version of the model may need to be inserted. It may be possible to arrive at this overlapping solution by disabling the packing function during molecular replacement.

3.4. Case studies

3.4.1. rsTagRFP

In 2014 Pletnev and co-workers [46] reported the structure of the reversible photoswitchable fluorescent protein rsTagRFP (PDB: 4KPI) which contains a form of ROD. They initially knew something might be strange with the structure when the diffraction pattern contained diffuse elongated reflections interspersed with sharp reflections (Figure 4). The sharp reflections were indexed and integrated, and the diffuse reflections were ignored. The integrated data scaled well to space group I422 with an R_{merge} of 0.046 (0.569).

The diffraction data were tested for twinning using XPREP [64], TRUNCATE [66] and the UCLA MBI Twin Detection server [68]. No twinning was observed (Figure 5). The

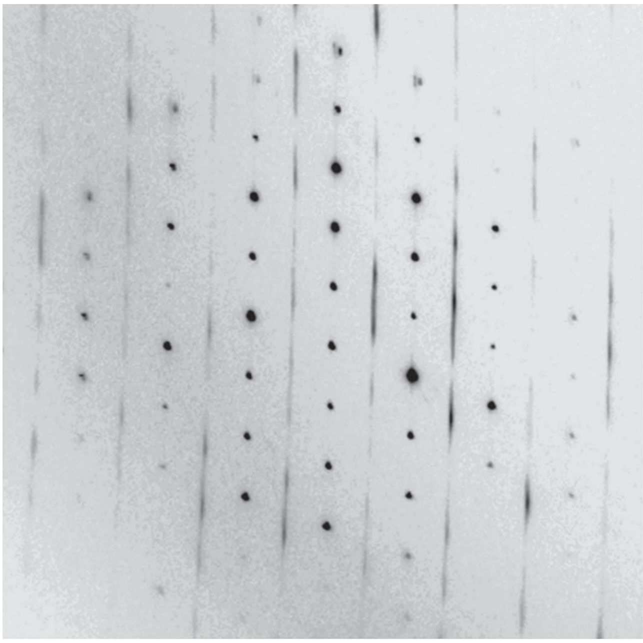

Figure 4. ROD diffraction pattern of rsTagRFP [46]. Reproduced with permission of the International Union of Crystallography.

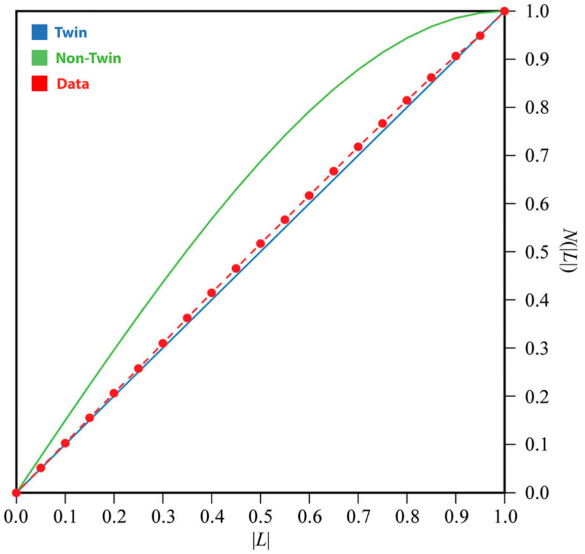

Figure 5. L test plot showing lines for a twin distribution and non-twin and the actual data [46]. Reproduced with permission of the International Union of Crystallography.

calculated V_m [86] returned an unreasonable value of 1.0 $Å^3Da^{-1}$, which would only allow one half of a monomer per asymmetric unit.

Molecular replacement was performed using the MOLREP programme [73] to find the initial structure. Due to the overlapping nature of the structure, the packing function

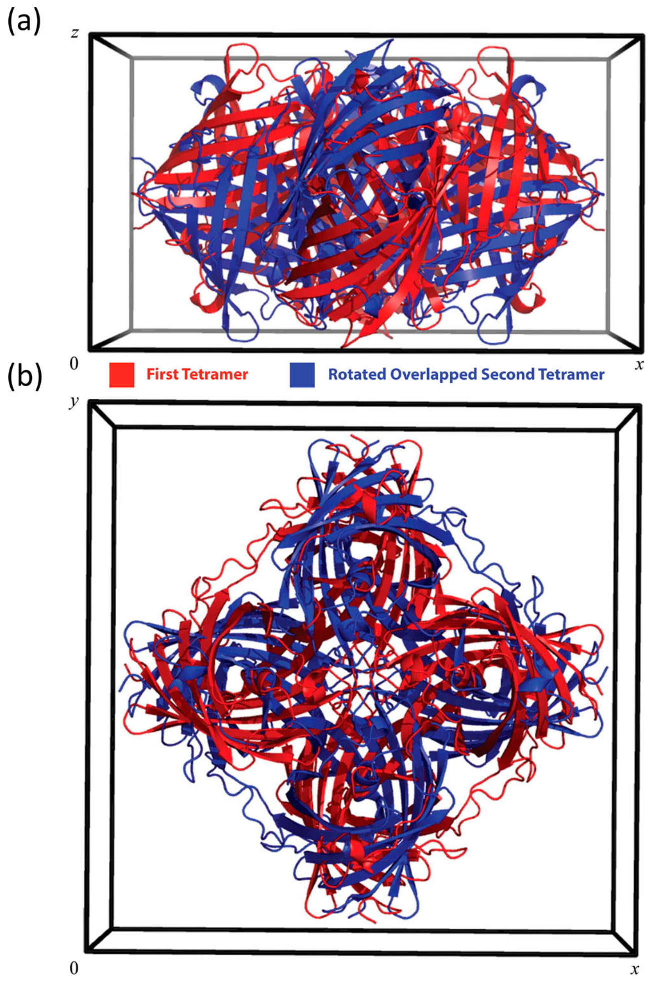

Figure 6. rsTagRFP model showing all of the symmetry-related chains coloured by tetramer [46] from the side (a) and the top (b). Reproduced with permission of the International Union of Crystallography.

option in MOLREP was disabled. Structure refinement was carried out with REFMAC5 [75], Coot [79], and phenix.refine [76]. Disabling repulsions for overlapping atoms during refinement cycles was also necessary. The final refinement is shown in Figure 6. The asymmetric unit consisted of a single molecule. After symmetry-related elements are generated there are two overlapping tetramers (Figure 6).

In order to validate the refinement, omit maps were generated for areas where the two structures overlap. An example is shown in Figure 7(a) where the omit map is shown with the two overlapped structures inserted. In Figure 7(b), the density has been coloured differently for each of the overlapping structures. In this crystal, the protein assembles into tetramers, and then these tetramers are incorporated into the crystal lattice. About half of the time the tetramers incorporate rotated 90 degrees relative to one another. For modelling purposes the authors assumed that process was random.

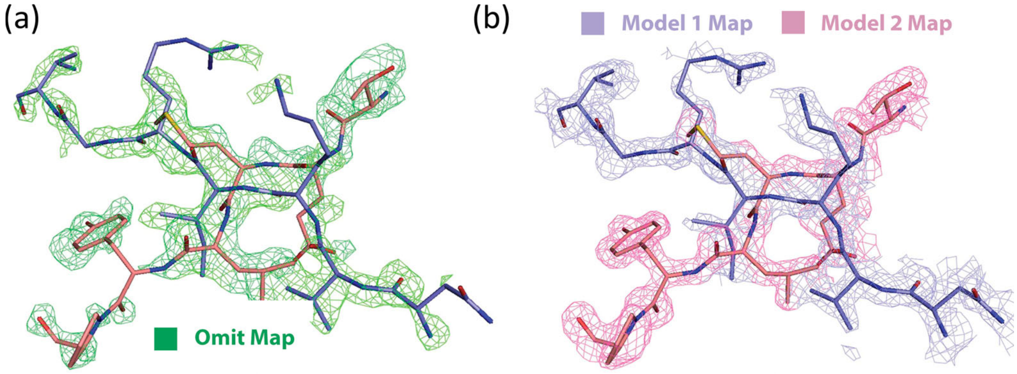

Figure 7. Refinement Results (a) omit map (green) with overlapping models inserted and (b) models with the overlapping density coloured by model [46]. Reproduced with permission of the International Union of Crystallography.

The authors go on to point out that due to the diffuse elongated reflections observed in the diffraction pattern that in actuality there may be some form of order to the way the tetramers assemble with a well-formed layer followed by a more disordered layer but that this aspect was not modelled.

3.4.2. Stefin B

Renko and colleagues [47] described the solution of an interesting crystal of stefin B (PDB: 4N6 V) which exhibited partial ROD in the crystal lattice. Stefin B is a protein thought to play a role in protecting against the proteases leaking from lysosomes. In this case, the diffraction pattern appeared normal (Figure 8) with no visible signs of the elongated diffuse reflections that have been observed for various types of order–disorder defects.

It was only after a careful analysis of the processing statistics and inspection of the electron density that the nature of this ROD structure was solved. In this case, they presented the processing of two crystals (crystal 1 and crystal 2). In both crystals, the reflections scaled well into several space groups (C2, F222, I4, and I422) with reasonable statistics. In the highest symmetry space group (I422), crystal 1 had a much higher number of reflections that were rejected and a doubling of the R_{merge} value. These observations in the statistics lead the authors to test for twinning and tNCS. With the largest off origin peak intensities less than 6% the origin peak there was no significant tNCS. The twinning analysis of both datasets (Figure 9) demonstrated that the reflections more closely match the nontwinned case versus the perfectly twinned case.

The structure was solved using Phaser [57] and refined with REFMAC5 [75], MAIN [77], and Coot [79]. The data were verified with phenix.xtriage [69]. Molecular replacement found four molecules in the asymmetric unit resulting in a four-layer stack of molecules (layers in the **a**-**b** plane with stacking in **c**) with a gap of no density on what would be the fifth layer to fill in the rest of the unit cell completely. The stacking along **c** is shown in Figure 10 with the 5th layer occupied by an overlapping structure. The authors had collected enough data to process everything in P1 which allowed them to place ten tetramers with the 9th and 10th tetramers occupying the previously empty layer in the unit cell. By inspecting the density around the 9th and 10th tetramers, it was possible to

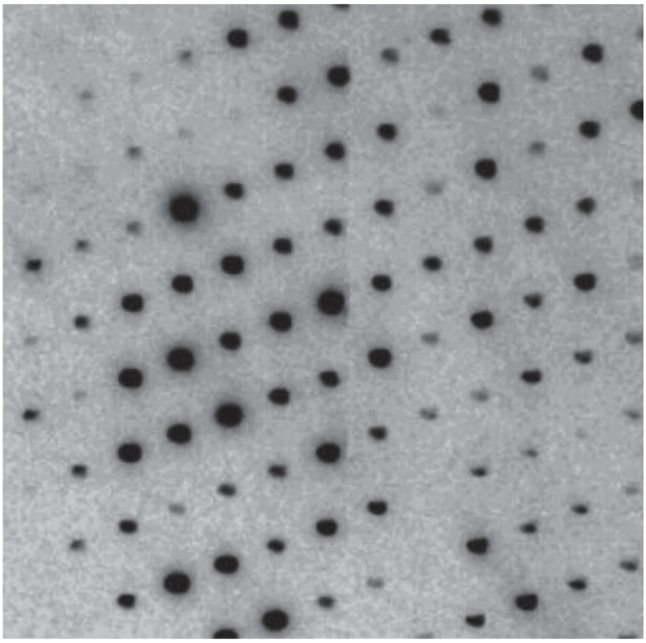

Figure 8. Stefin B diffraction pattern [47]. Reproduced with permission of the International Union of Crystallography.

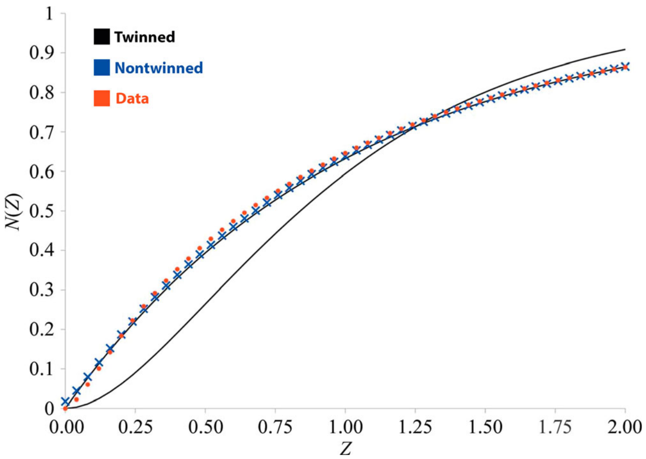

Figure 9. Cumulative intensity test plot for the Stefin B reflections. The solid line represents the case for twinned data. The 'x' line nontwinned and the dots the reflection data that was observed for stefin B [47]. Reproduced with permission of the International Union of Crystallography.

see evidence for a rotated overlapping tetramer [47]. The electron density fits with the overlapping model quite nicely (Figure 11). After reviewing the model the correct spacegroup was determined to be I4.

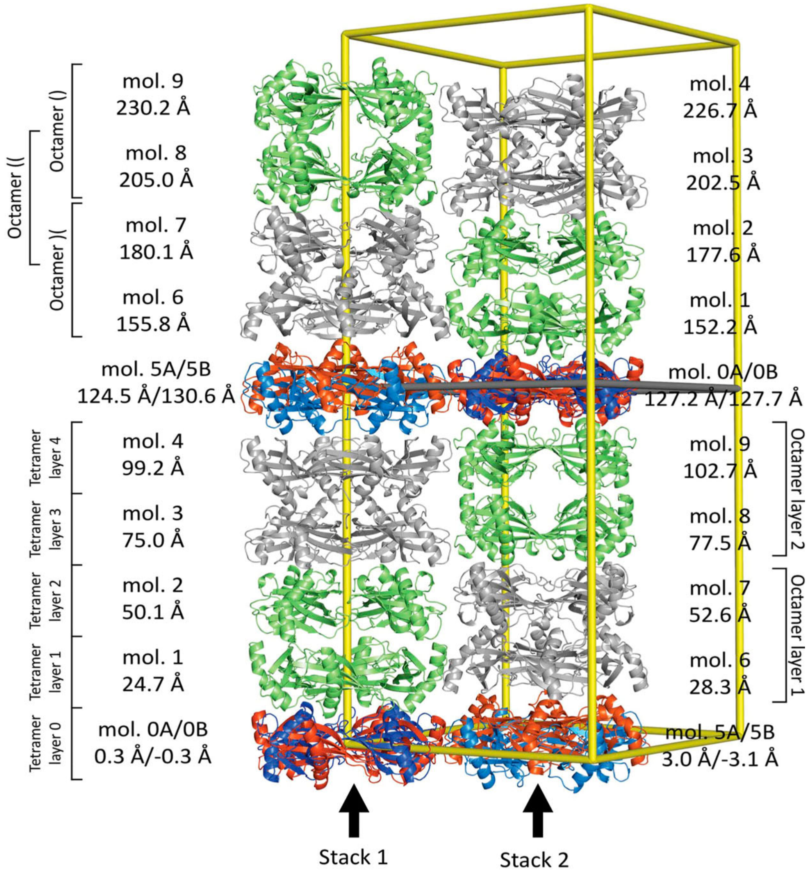

Figure 10. Stefin B model showing ROD layer every 5th tetramer in the stack [47]. Reproduced with permission of the International Union of Crystallography.

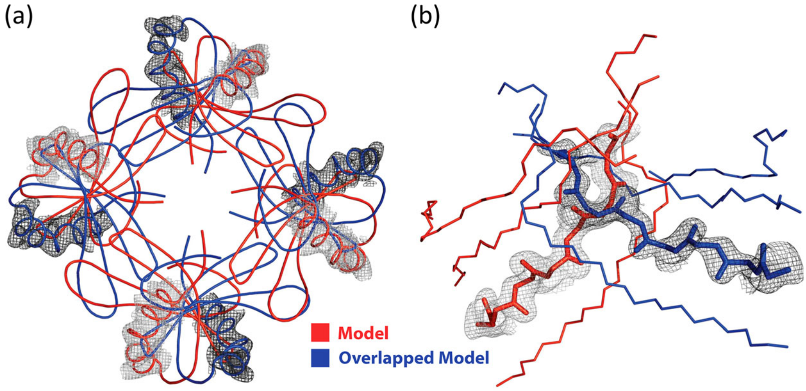

Figure 11. Stefin B OMIT electron density maps around the overlapped regions with models overlaid [47]. Reproduced with permission of the International Union of Crystallography.

4. Lattice-translocation defects (LTD)

4.1. What is it?

Lattice-Translocation Defects are a form of crystal disorder where some layers of the crystal are displaced relative to the layer above or below. These dislocated layers are well ordered (Figure 12). The solution is to deconvolute the effects caused by the translocations from the reflection intensities.

4.2. Symptoms

LTD manifests itself in the diffraction pattern as groups of reflections that are streaky along with other groups of reflections that are well defined. Figure 13(a) shows a diffraction pattern from a crystal of Lens Epithelium Derived Growth Factor (LEDGF) [44] with an area of strong sharp reflections as well as areas of streaky reflections. Another way to detect this phenomenon is to look at the Patterson map. For the LEDGF case, the Patterson map has a peak at $u = 0.1$, $w = 0.1$ (Figure 13(b)) which represents a spacing that is not possible for tNCS because that distance is too small to be an adjacent molecule. It is also possible to see ghost density in the molecular replacement solution (not shown).

4.3. Solution

The approach that is used to solve the LTD problem is to correct the reflection intensities to be as if there was no LTD. Different approaches [36,38,39,44,88] have been proposed to

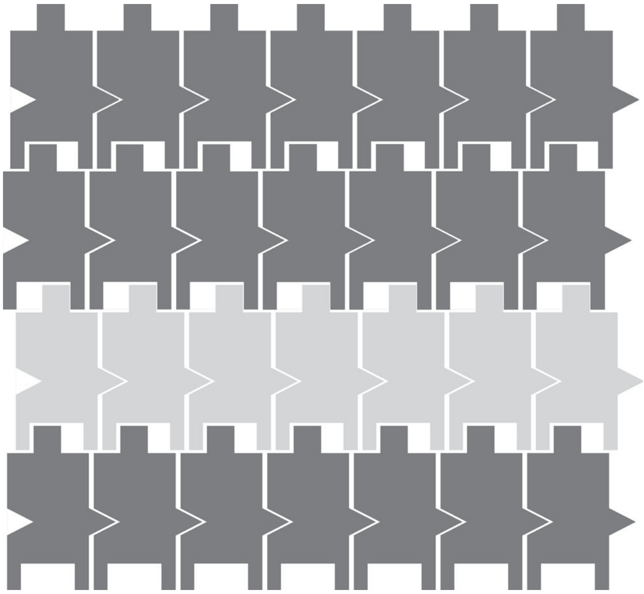

Figure 12. Example of an LTD defect. The layers assemble left to right reliably. In the top to bottom stacking of the layers, it is possible for random layers to be shifted to the left or right occupying one of two possible positions (denoted by different colours).

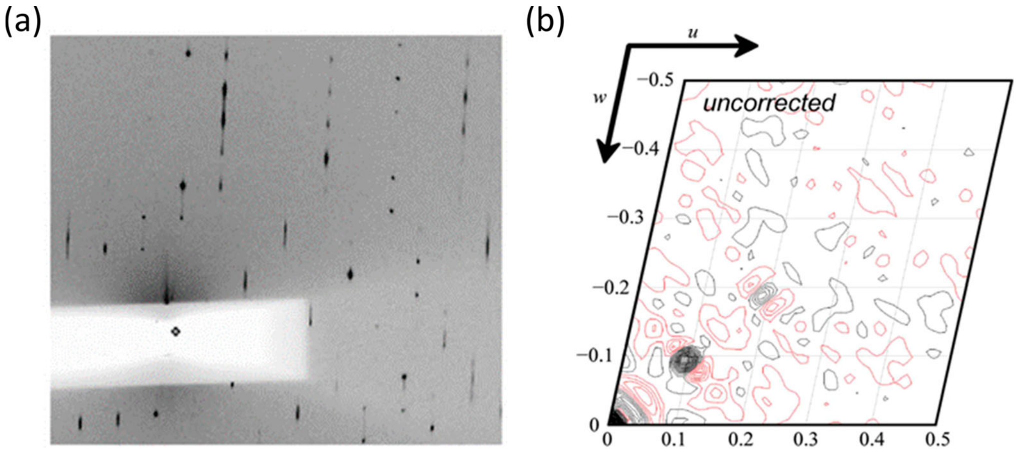

Figure 13. LTD example showing (a) diffraction pattern with sharp and streaky reflections and (b) Patterson map with strong off origin peaks at a spacing that cannot be tNCS [44]. Reproduced with permission of the International Union of Crystallography.

correct the intensities. Two case studies will be presented in detail below demonstrating two different ways to perform the correction [44,88].

4.4. Case studies

4.4.1. MVV $IN_{NTD+CCD}$–$LEDGF_{IBD}$ complex

Wang and co-workers [38] first proposed an LTD correction in 2005. This case study will focus on their 2009 paper [44] that provides a method to determine the translocation fraction which is critical to perform the correction. This automated translocation fraction approach represents an improvement to the original LTD correction. The correction was applied to diffraction data from a complex of Maedi-Visna Virus (MVV) Integrase (IN) truncation (MVV $IN_{NTD+CCD}$) and a truncation of LEDGF ($LEDGF_{IBD}$) or MVV $IN_{NTD+CCD}$–$LEDGF_{IBD}$ Complex (PDB: 3HPG) which will just be referred to as LEDGF Complex for the rest of this section.

The simplest model of LTD is a layer displacement where the following equation can describe the effect on intensity

$$I_{\text{total}} = f I_{\text{unit}} \tag{1}$$

where

$$f = A[1 + r\cos(2\pi \mathbf{h}\mathbf{t}_{\mathbf{d}})] \tag{2}$$

$$A = 2k^2 - 2k + 1 \tag{3}$$

$$r = 1 + \frac{2k(1-k)}{2k^2 - 2k + 1} \tag{4}$$

Where, f is a scale factor, $\mathbf{h}$ is a reciprocal space vector (hkl), $\mathbf{t}_{\mathbf{d}}$ is a translocation defect vector, and k is the fraction of translocation defects. Equations 1–4 show that if the k fraction of dislocation defects can be determined, then the measured intensities (I_{total}) can be

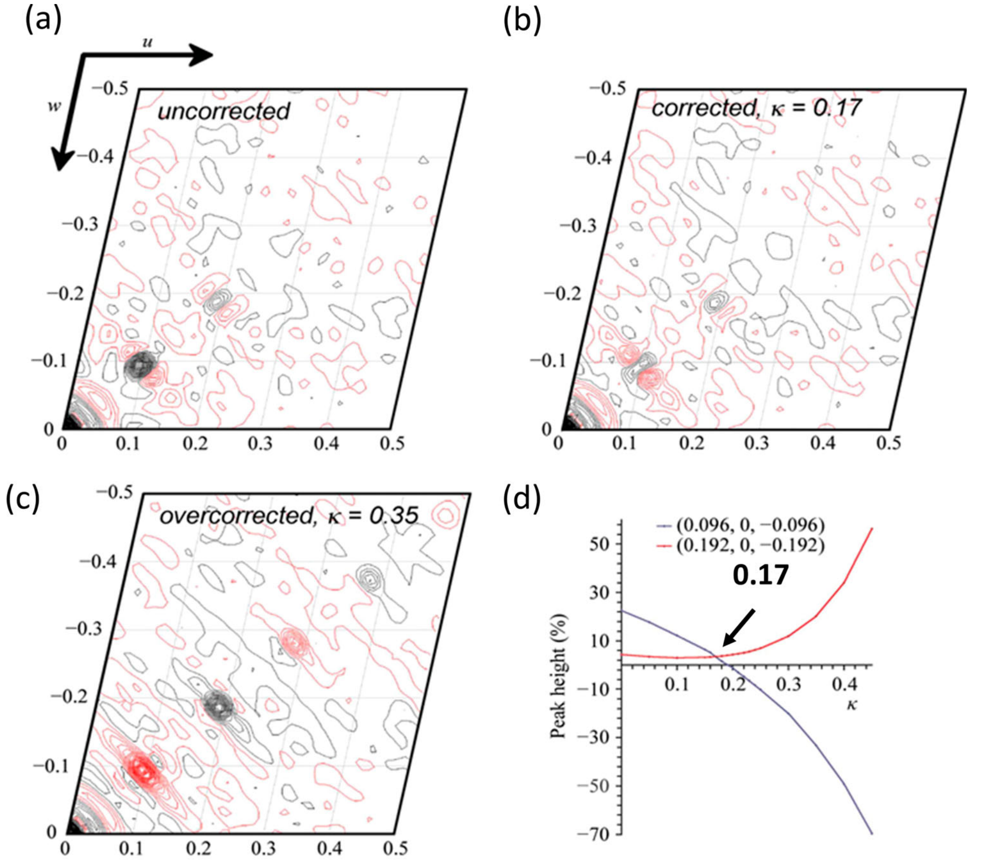

Figure 14. LEDGF Patterson map as Function of k fraction (a) Native (b) $k = 0.17$ (c) $k = 0.35$ (d) LTD Patterson Peak vs Ghost LTD Patterson Peak due to the correction as a function of k fraction [44]. Reproduced with permission of the International Union of Crystallography.

divided by the scale factor (f) to arrive at the corrected intensity (I_{unit}). One way to determine k is to monitor the Patterson map as a function of k as shown in Figure 14. There is an optimum position where the undesired Patterson peak from the translocation defect is minimized (Figure 14(b)) while at the same time also minimizing other negative Patterson peaks that may appear when the intensities are overcorrected (Figure 14(c)). From the plot of peak height, the best value for k is around 0.17 for this sample (Figure 14(d)).

In the LEDGF complex paper [44] two crystals are discussed. One crystal had a normal-looking, sharp diffraction pattern while the other had sharp reflections interspersed with streaky ones (Figure 15(a) and (b)). When the average intensity plot for different layers was made, it was clear that both crystals are suffering from the same sort of translocation defect but one sample had more of it (Figure 15(c)). Using the corrective approach outlined above the corrected intensities are shown for different values of k for both data sets (Figure 16).

For the dataset that had the highest amount of modulation in the intensity of the reflections the best value of k for the correction was $k = 0.17$ (Figure 16(a)). For the case where the observed LTD (based on layer average intensity) had the lower peak to peak distance,

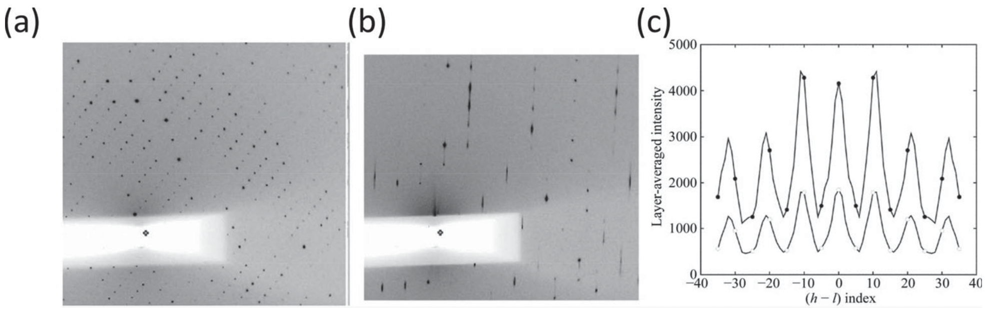

Figure 15. LEDGF complex diffraction from two Samples. (a) Minor LTD that was not visible in the diffraction pattern (b) More severe LTD that was easily seen in the diffraction pattern (c) layer averaged intensity for reflections that satisfy the *h-l* condition (for example, all the intensity of all of the reflections where *h-l* = −10 was about ∼4300 for one crystal and 1800 for the other crystal) [44]. Reproduced with permission of the International Union of Crystallography.

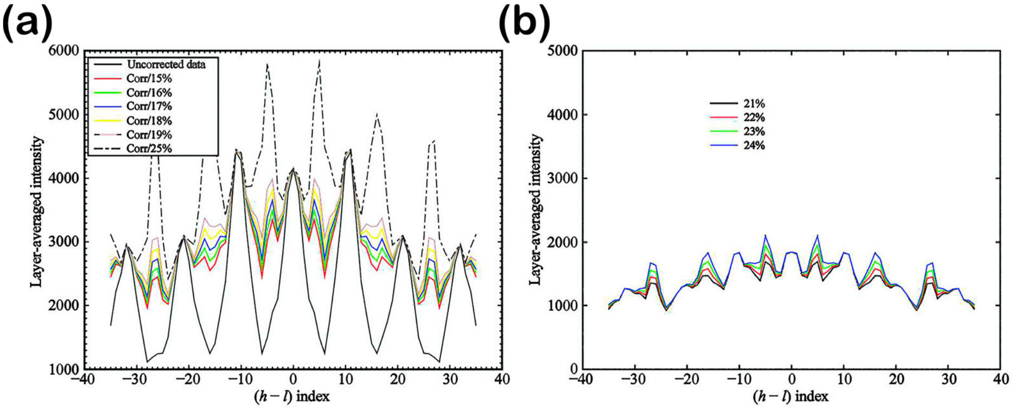

Figure 16. LTD Intensity Correction as a function of *k* for two samples (a) Visible LTD in the diffraction pattern (Figure 15(b)) no LTD in the diffraction pattern (Figure 15(a)) [44]. Reproduced with permission of the International Union of Crystallography.

the best value of *k* was around 0.22 (Figure 16(b)). These values of *k* provided a good balance between equalizing the intensity of the reflections to minimize the strong LTD related Patterson peak while at the same time preventing a sizeable negative peak that could be caused by overcorrection.

The paper was very well put together, and easy to follow, but could have been made even better, in our opinion, had they included a before and after electron density map to show the effect the correction had on the quality of the electron density. In this case, the reader is left to observe the power of the correction from the improvement in the statistics for the refinement which show good improvement with R and R_{free} being reduced from 27% and 30% to 22.5% and 25%, respectively. What is both interesting and little scary about this paper is that the translocation defect is present even in the dataset that appeared to have ‘normal’ diffraction (Figure 15(a)) which leads to the conclusion that it is possible this problem could be more wide spread but just undetected.

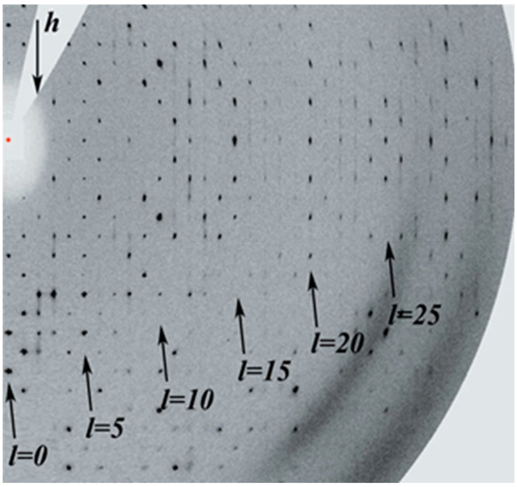

Figure 17. CRMP-4 diffraction pattern showing streaky diffuse reflections interspersed with strong sharp reflections [88]. Reproduced with permission of the International Union of Crystallography.

4.4.2. CRMP-4

An alternate approach to correcting reflection intensities where translocation defects were observed in the diffraction pattern has been proposed by Ponnusamy and colleagues [88]. As shown in the diffraction pattern of Collapsin Response Mediator Protein-4 (CRMP-4) crystal, there are streaky diffuse reflections interspersed with strong, sharp reflections (Figure 17). This type of diffraction is an indication that the sample has some OD issue, with the most probable being LTD.

The foundation of their approach centres around the idea that the crystal is composed of perfect layers and occasionally one of these layers is dislocated by an amount described by a translocation vector ($\mathbf{t_d}$). From the $\mathbf{t_d}$, all dislocations can be described as either a positive or negative offset $\mathbf{t_d}$ relative to the lattice. This offset leads to the following two equations

$$I_o = I_c[q_0 + q_1 \cos(x) + q_2 \cos(2x) + \cdots]/[q_0 + q_1 \cos(x)] \tag{5}$$

where

$$x = \pi(h + k + l + \tau l) \tag{6}$$

Here, I_o is the observed intensity of a reflection (crystal plus defect), I_c is the intensity for the fraction of the sample that is part of the crystal, and τl is related to the translocation vector. τl accounts for the translocation component $\mathbf{t_d}$ because in the CRMP-4 example the translocation vector is along the **c** direction or l in reciprocal space. Equation 5 demonstrates that there is a complex nonlinear scaling factor for observed reflection intensity. Further by combining it with Equation 6 there are groups of reflections (those with the same value of $(h + k)$ and the same l) which will have the same scale factor because they have the same value of x. From these groups of reflections that should have the same scale

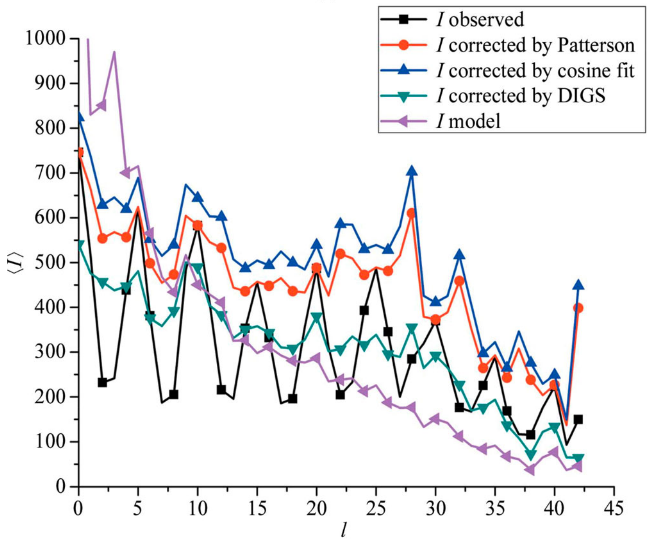

Figure 18. CRMP-4 original intensities versus corrected intensities using different LTD correction approaches [88]. Reproduced with permission of the International Union of Crystallography.

factor leads to the following equation

$$F_o^{(n)} = \left(\sum F_c^{(n)} / \sum F_o\right) F_o \qquad (7)$$

Equation 7 provides the basis for their demodulation method called DIGS (Demodulation of Intensities by Grouping and Scaling) where through an iterative process (current iteration is n), reflections within the same group (Σ) are scaled to a new observed structure factor (${F_o}^{(n)}$) by using a scale factor. The scale factor is determined by calculating new structure factors in the reflection group ($\Sigma {F_c}^{(n)}$) and dividing by the observed structure factors (ΣF_o) in the group. This process is repeated iteratively until convergence is reached. For CRMP-4 convergence was reached in 2–5 cycles of REFMAC5 [75] refinement to generate the model used for structure factor calculations.

Using DIGS provided about a 5% improvement in R and R_{free} over the uncorrected data and about a 1% improvement over the Wang approach. Figure 18 shows electron density plots of the observed intensities, as well as with corrected intensities for the Patterson, cosine fit / least squares [36], DIGS approaches. Visually the DIGS approach seems to be slightly better than cosine function and Patterson flattening (Figure 19(a–d)).

5. Translational non-crystallographic symmetry (tNCS)

5.1. What is it?

Problematic tNCS results when there are large numbers of repeating components (> 3) in the asymmetric unit. For example, this repeating component could be several copies of a subunit or chain. These extra copies are described by a translation vector between adjoining tNCS molecules. Proteins are flexible, and often this tNCS represents some modulation within the crystal where there can be a range of conformations that the components occupy.

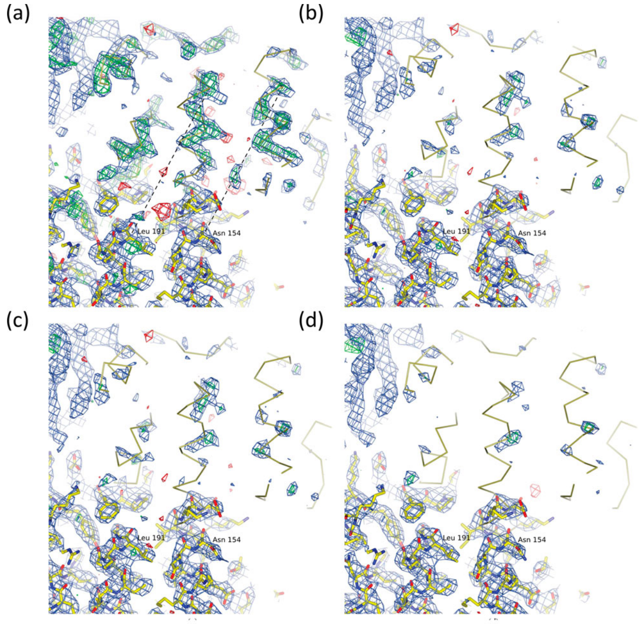

Figure 19. CRMP-4 electron density (a) uncorrected showing LTD ghost density (dashed lines showing the translation to ghost helices) and then LTD corrected (reduced density for the ghost helices) with (b) cosine function, (c) Wang's Patterson flattening, and (d) DIGS [88]. Reproduced with permission of the International Union of Crystallography.

tNCS results in a modulation in the measured reflection intensities. Depending on the amount of disorder associated with the tNCS, software programmes may have difficulty determining the proper spacegroup and/or performing molecular replacement effectively. The user may have to enter the tNCS description manually in order for the software to use it properly.

5.2. Symptoms

Molecular replacement fails. The easiest way to detect tNCS is to investigate the Patterson map and look for strong peaks. The location of these strong peaks can be used to determine the number of tNCS molecules and how they are distributed in the asymmetric unit cell. Peaks in the Patterson map that are indicative of tNCS have spacing that matches the size of the macromolecule. Peaks with spacing other than macromolecular spacing could indicate LTD. There may also be issues with space group ambiguity where multiple space groups appear as potential candidates, and it is difficult to sort out the correct one.

5.3. Solution

Typically tNCS is handled by molecular replacement software. In some cases, it is handled automatically or may require user intervention (e.g. by entering by hand the vector that describes the translation). The software attempts to estimate the tNCS effect by analyzing the intensities and modelling the modulation observed in the intensities using the tNCS

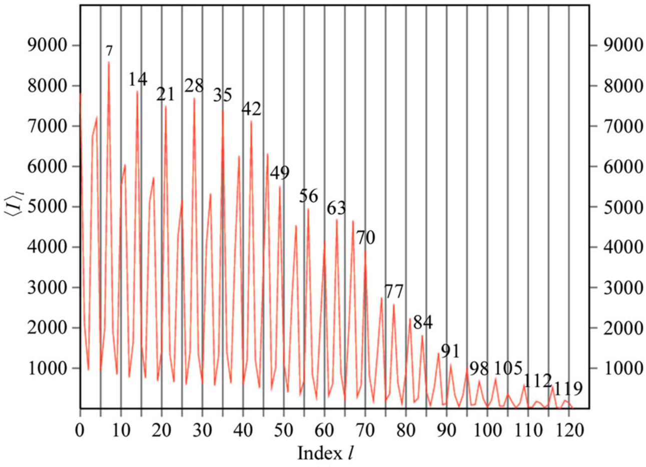

Figure 20. Hyp-1-ANS mean reflection intensity versus index l [56]. Reproduced with permission of the International Union of Crystallography.

vector plus small deviations in translation and rotation. tNCS modelling of the modulation of the intensities is done before the molecular replacement occurs to improve the chances of finding the correct solution.

5.4. Case studies

5.4.1. Hyp-1-ANS

Large crystals of pathogenesis-related class 10 protein (Hyp-1) in a complex with 8-anilino-1-naphthalene sulfonate (ANS), or the Hyp-1-ANS complex, were obtained and diffraction data were collected to 2.4 Å resolution [56]. The initial data merging appeared to be satisfactory in the P422 space group. There was an existing Hyp-1 structure available for use with molecular replacement. Repeated attempts at molecular replacement failed.

Further study of the reflections provided some interesting results. The intensity of the reflections was modulated where there was a maximum when the *l* index was divisible by 7 (Figure 20). After observing the modulation pattern in the mean reflection intensity the authors decided to inspect the Patterson Map.

The Patterson maps showed strong peaks relative to the origin with a spacing of about 1/7 (Figure 21) in *w*. The highest off-axis peak was about 72% of the origin, and it was located at about 2/7*w*. These peaks are characteristic of tNCS. In order to get Phaser [57] to properly lock onto the right solution, it became necessary to add tNCS modelling.

The effects of tNCS can be modelled into the modulation observed in the intensities by assuming that tNCS component is a close match but with some small amount of translation and rotation relative to the other tNCS molecules. The effect on phase and summed intensity can be calculated for a perfect tNCS molecule and then using some mathematical simplifications and probability distributions, the effects caused by permutations from the ideal case can be accounted. The theory and application are spelled out mathematically in [56]. After Phaser [57]was adjusted to include this more complete model of tNCS, molecular replacement was attempted in the P422 point group. There were several solutions discovered, but none of them was successful on further investigation. The thought

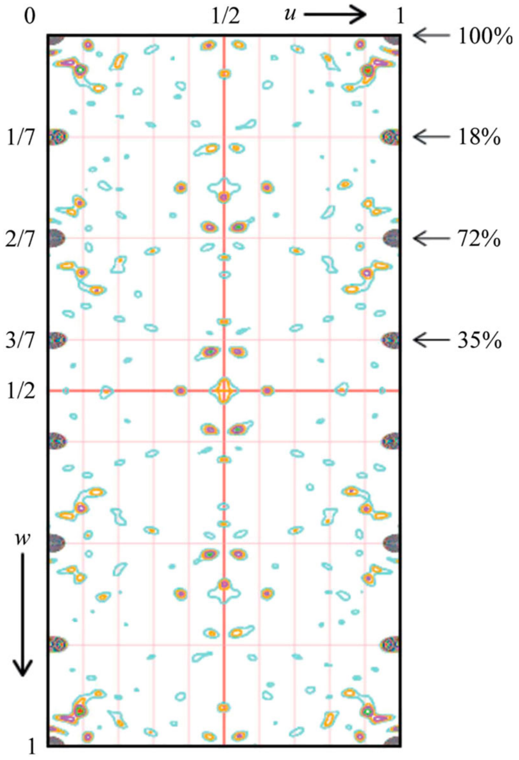

Figure 21. Patterson Map Section [35] with $u = 0$ [56]. Reproduced with permission of the International Union of Crystallography.

was that although the reflections processed reasonably well in P422 that the actual point group was something with lower symmetry.

The solution was to solve the molecular replacement problem in P1. First, the reflections were expanded to P1 and then the search was done by using a 7x assembly of Hyp-1 where molecules 2–7 were positioned relative to molecule one by using the translocation vector (−0.004, −0.004, 0.285) which is derived from the Patterson map (Figure 21). Rigid body refinement was used to improve the fit of these 7 molecules. Next, seven additional copies of this refined ensemble were fit into the P1 cell with Phaser [57], resulting in 56 Hyp-1 monomers (8 ensembles consisting of 7 monomers each) in the final solution.

Analysis of this P1 solution revealed that the correct space group was C2 (Figure 22) by observing symmetry in the P1 space group. An additional problem was that the data collection strategy used was based on the P422 space group that was initially identified, so the end completeness (72%, Table 1) was not as high as desired. The data statistics for C2 space group shows an improvement in R_{merge} from 7.5–6.6%.

5.4.2. FBPase

Fructose-1,6-bisphosphatase (FBPase) with bound adenosine monophosphate (AMP) in the T (inactive) state resulted in a crystal with four possible space groups $P4_122$, $P4_322$, $P4_12_12$ and $P4_32_12$ [89]. For this crystal, tNCS related elements resulted in a unit cell that was 3x longer in the c dimension than what may have been initially estimated. The

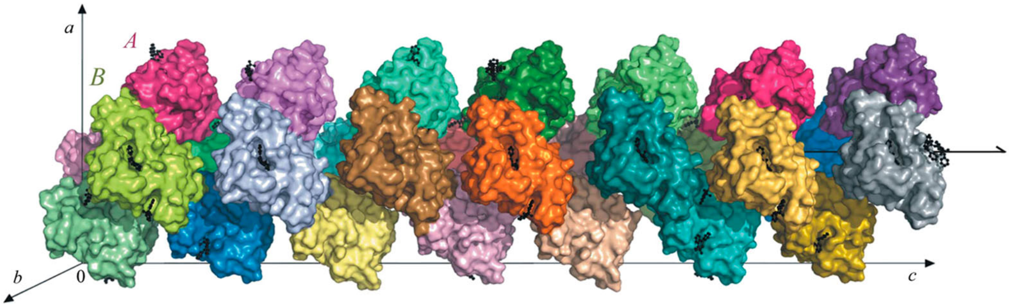

Figure 22. 28 Molecules in the Hyp-1-ANS asymmetric unit cell [56]. Reproduced with permission of the International Union of Crystallography.

Table 1. Statistics for Hyp-1-ANS structure [56]. Reproduced with permission of the International Union of Crystallography.

Beamline	19ID, SER-CAT, APS	
Temperature (K)	100	
Space group	*P*422	*C*2
Unit-cell parameters		
a (Å)	103.42	146.21
b (Å)	103.42	146.12
c (Å)	298.50	298.35
β (°)	90	90.07
Wavelength (Å)	1.000	1.000
Resolution (Å)	30–2.43 (2.47–2.43)	30–2.43 (2.47–2.43)
Reflections, measured	496,579	495,931
Reflections, unique	61,810	107,447
Completeness (%)	99.8 (99.2)	72.7 (65.9)
$I/\sigma(I)$	26.4 (2.6)	13.4 (1.5)
R_{merge}[a] (%)	7.5 (75.8)	6.6 (69.1)
Multiplicity	8.0 (7.1)	2.9 (2.6)

[a] $R_{merge} = \sum_{hkl} \sum_i |I_i(hkl) - I(hkl)| / \sum_{hkl} \sum_i I_i(hkl)$.

increase in c is because when AMP binds to FBPase, there is a rotation in the tetramer (Figure 23). The AMP induced conformational change breaks the $I4_122$ symmetry that would have existed and which would have allowed for a 3x smaller unit cell and a much simpler molecular replacement solution.

Investigation of the Patterson map showed multiple large peaks that indicated (Figure 24) the presence of tNCS. Whenever something unexpected appears it is always a good practice to study the reflection intensity analysis to make sure that the data is not twinned. In this case, the intensities are consistent with data that is not twinned (Figure 25).

Molecular replacement found solutions for all of the possible P422 space groups (Figure 26(b–e)). The correct solution was $P4_12_12$ because overall it had the best statistics with R and R_{free} of 0.21 and 0.25, respectively. The other solutions had R/R_{free} of 0.22/0.25 for $P4_122$, 0.25/0.28 for $P4_322$, and 0.24/0.28 for $P4_32_12$. On closer inspection, all of the solutions were just variations on the same theme. If the $P4_12_12$ solution is rearranged from Figure 26(a) to (b) it is much easier to see how the other solutions (Figure 26(c–e)) are related to the $P4_12_12$ solution. The $P4_32_12$ solution is related through a translation of $-1/3c$

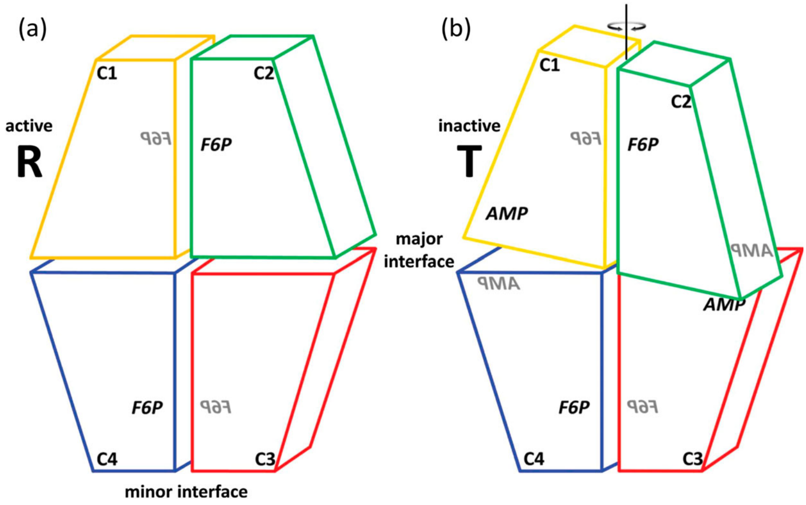

Figure 23. FBPase Schematic showing the active (a) and inactive AMP bound form (b) [89]. Reproduced with permission of the International Union of Crystallography.

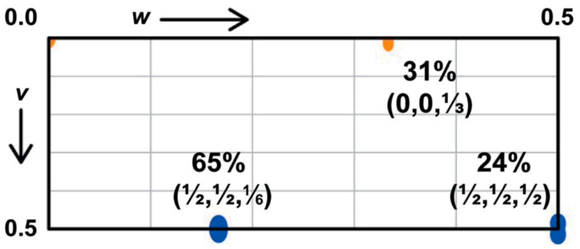

Figure 24. Patterson Map of FBPase [89]. Peaks coordinates are (*u*,*v*,*w*) where the *u* dimension is coming out of the page for positive *u*. Reproduced with permission of the International Union of Crystallography.

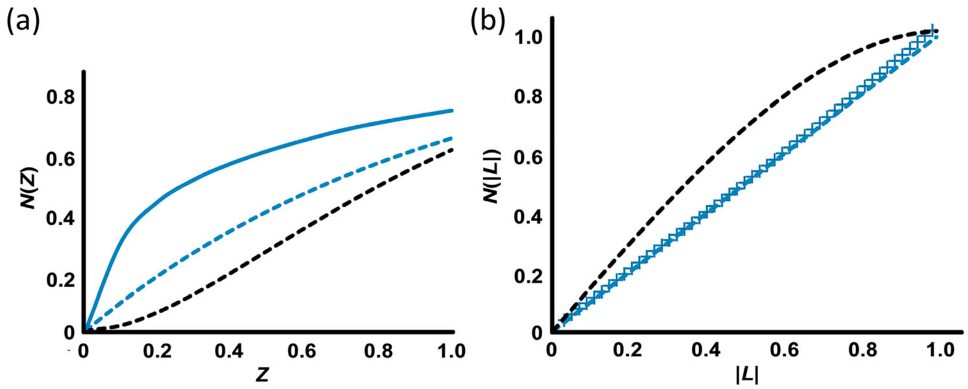

Figure 25. Reflection statistics for twin analysis. (a) Cumulative intensity distribution of acentric reflection where black dashed line is for the twinned case and the other dashed line is for the untwinned case and the solid line represents the actual data. (b) L-test where the black dashed line is for the twinned case, the dashed line is for the untwinned case and the *x*'s represent the data. Both approaches indicate that the data are untwinned [89]. Reproduced with permission of the International Union of Crystallography.

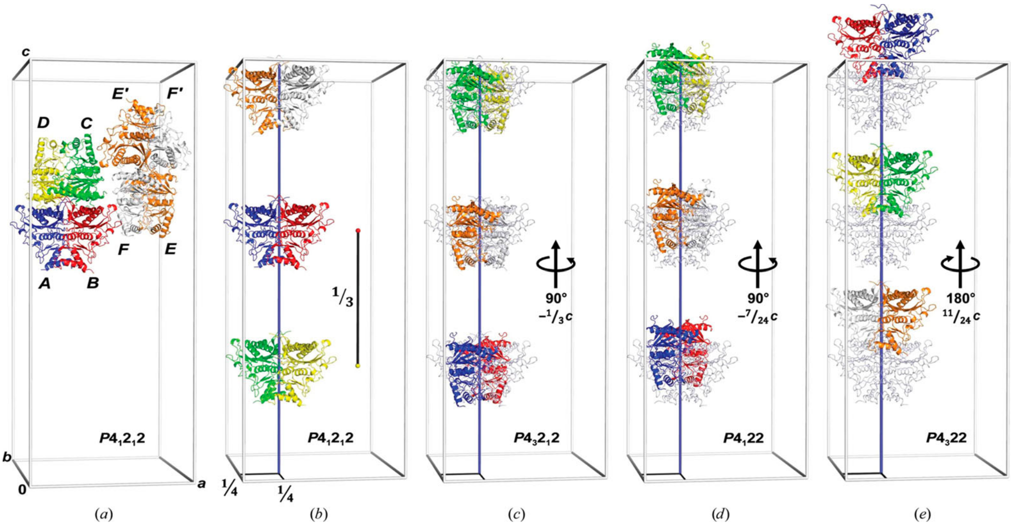

Figure 26. Possible molecular replacement solutions for FBPase with AMP bound showing how all of the solutions are related to each other through (a) $P4_12_12$ and (b) alt $P4_12_12$, with alt $P4_12_12$ in grey overlaid on the (c) $P4_32_12$, (d) $P4_122$ and (e) $P4_322$ solutions [89]. Reproduced with permission of the International Union of Crystallography.

and a rotation of 90° about an axis along c passing through ¼a, ¼b. For the $P4_122$ case, the shift is −7/24c and a rotation of 90° at the axis along c again passing through ¼a, ¼b. For the $P4_322$ case, the shift is 11/24c and a rotation of 180° along that same axis about c.

6. Incommensurately modulated crystals

6.1. What is it?

Situations arise in which a periodic modulation overlays itself on the crystal lattice. Two categories of these types of modulations have been observed. The first is known as a displacement modulation where atoms move relative to some average position from one unit cell to another unit cell. The other possibility is an occupational modulation where an atom may be replaced with another atom or may be absent in some unit cells. In both cases, the underlying mechanism that created this modulation is periodic. There are two categories of periodic modulations. Commensurate modulations have an underlying phenomena that has a period that is equal to an integer number of unit cells. The other category is incommensurate where an integer number of unit cells do not line up with the modulation period. Figure 27 shows a one-dimensional displacement modulation with the modulation function overlaid on the unit cells [49]. In the periodic case, the protein is in the same position in consecutive unit cells (Figure 27(a)). In the commensurately modulated case, the protein oscillates between four states every four unit cells resulting in a 4x supercell (Figure 27(b)). In the incommensurate case, there are no repeated states in any of the consecutive unit cells (Figure 27(c)).

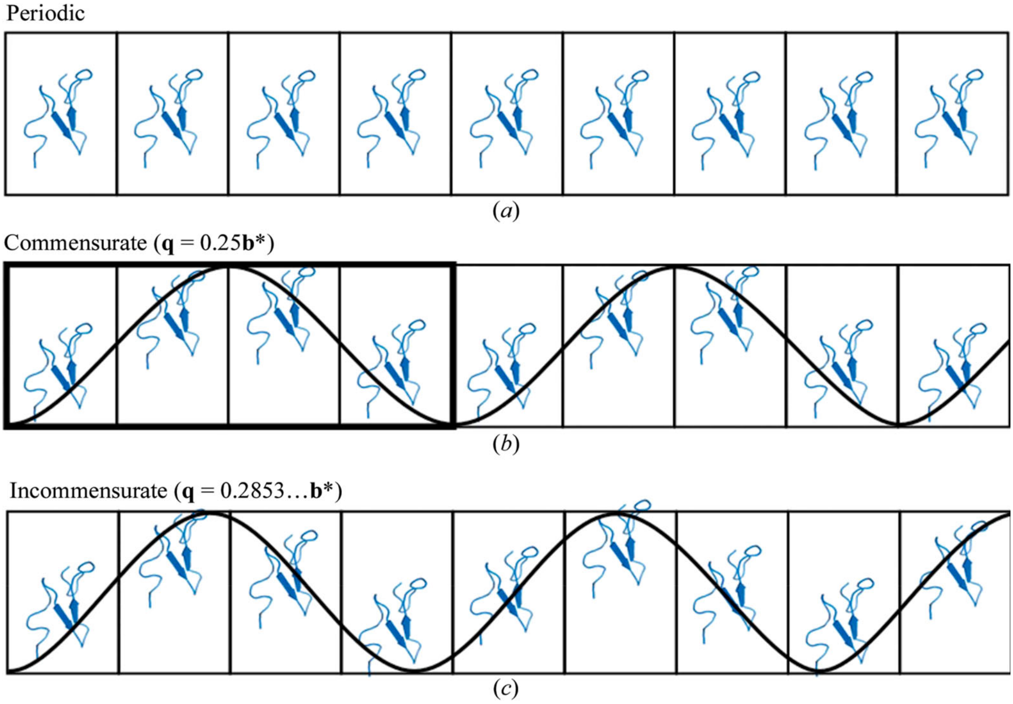

Figure 27. Example of how a one dimensional displacement modulation can be applied to (a) periodic unit cells to produce a (b) commensurate modulation or a (c) incommensurate modulation [49]. Reproduced with permission of the International Union of Crystallography.

6.2. Symptoms

Modulated crystals have a unique diffraction pattern with strong main reflections and weaker satellite reflections associated with the mains. It is the presence of sharp satellite reflections that prove the modulation is periodic. Figure 28 shows a diagram of a hypothetical incommensurate diffraction pattern with four main reflections and first- and second-order satellite reflections on either side of each main.

6.3. Solution

Currently the structure from an incommensurately modulated macromolecular crystal has not been accomplished because the software has not yet been adapted to handle this case. Several levels of modification of various software packages and core file storage formats will be needed. A few examples include adjusting the PDB format and supporting programmes to understand modulated structures, changing MTZ format and supporting programmes to include the superspace groups, adjusting the refinement programmes to work in n dimensions instead of just three. Incommensurately modulated small molecule structures are routinely solved with Jana2006 [74] and SuperFlip [78].

For macromolecules, one of the first challenges is to index and integrate the reflections using higher dimensions. The accepted approach is to define a **q** vector to describe the spacing of the satellite reflections relative to their main reflection (Figure 28). Using a **q**

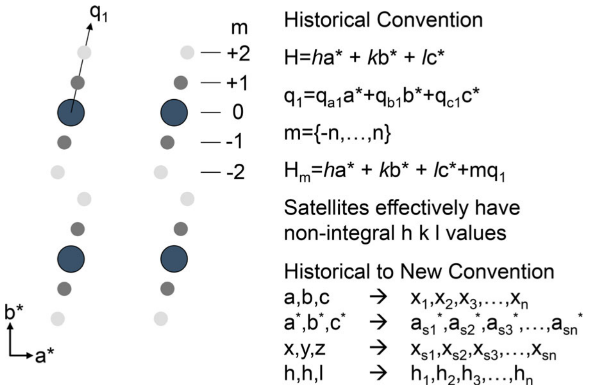

Figure 28. Incommensurate diffraction pattern schematic showing how a modulated diffraction pattern can be indexed using higher dimensions, in this case four (h, k, l, m). Depending on the age of an incommensurate publication it may represent the information as shown in the historical convention or the new convention.

vector leads to a four-dimensional (4D) indexing scheme where reflections have indices h, k, l and m for the case where the modulated reflections can be described with a single $\mathbf{q}$ vector. This type of indexing is known as (3 + 1)D because in reality only a smaller subgroup of the actual 4D space is used. Two notations describe the higher dimensional indexing, and both may be encountered in the literature. In the more classical extension, the indices are labelled as h, k, l, and m. In the newer more easily extensible description the h, k, l, and m are replaced with h_1, h_2, h_3, ... , h_n in reciprocal space and a, b, c, are replaced with x_1, x_2, x_3, ... , x_n as needed in superspace. Eval15 has been used to successfully index and integrate incommensurate protein crystals [49]. Once indexed, molecular replacement and refinement remain challenging. If the modulation is commensurate ($\mathbf{q}$ vector can be rationalized), then a supercell can be used to perform molecular replacement and refine the structure. The modulation will appear as a tNCS, and proper settings will need to be configured in the molecular replacement software. Another option is to fit an average structure to the main reflections and then perform refinement on a supercell expansion of the average structure. Currently, for incommensurately modulated samples the only option is to use a supercell approximation because of software limitations.

Satellite reflections are evidence of a periodic modulation that has overlaid itself on the crystal. The difficulty is how can atomic displacements that appear to be random in real space be periodic? The solution (Figure 29) shows how a periodic function in the extra dimension ($\mathbf{a}_{s4}$) can be used to describe the variation of the atomic position of an individual atom in 3D space ($\mathbf{R}$). Value of t along $\mathbf{a}_{s4}$ shows how many periods of the modulation function have occurred. The modulation function is periodic, so equivalent positions can be projected from other t values into the range 0–1 as shown in the zoomed-in region (Figure 29). The numbers along the right show how unit cells are encountered while travelling from 0 to 1 in t which is not in the same order they are traversed in real space. For this example, where there are two modulation waves every 7 unit cells, the unit cells are ordered as 1, 5, 2, 6, 3, 7, and 4 as t goes from 0 to 1. This reordering can be important in refinement if the modulation is known to be smooth (only first-order satellites observed) for example and during refinement, the atomic displacements in superspace are forced to be smooth

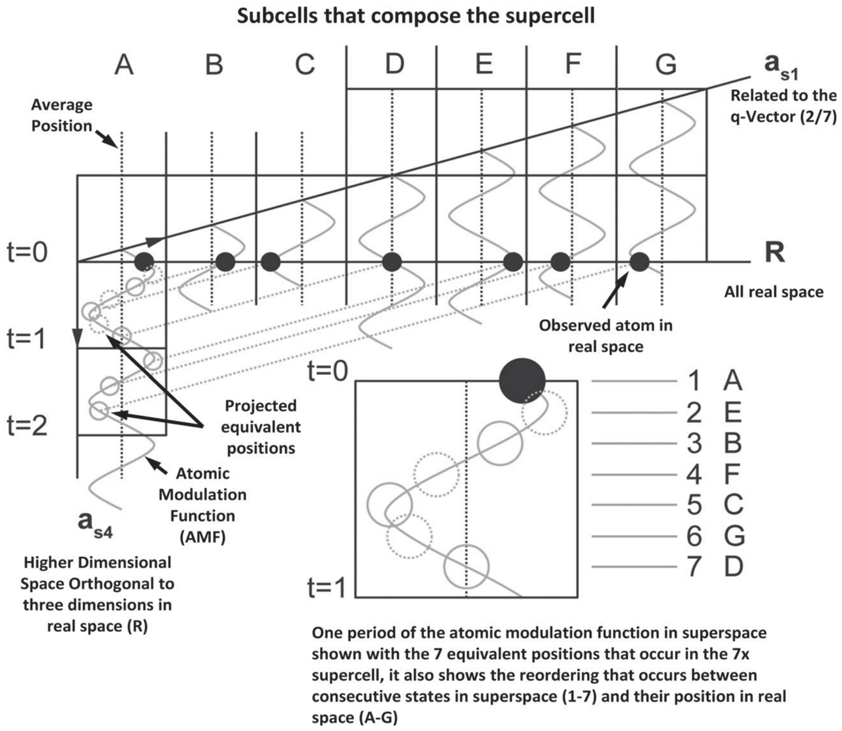

Figure 29. Superspace diagram showing how higher dimensional space can return periodicity to real space displacements that appear to be random in 3D space (**R**) [71]. The atomic modulation function (AMF) in the $\mathbf{a}_{s4}$ direction translates along the $\mathbf{a}_{s1}$ direction (related to the q-vector) to produce atomic positions (black dots) in real space where the AMF intersects real space. The AMF is periodic and because of equivalent positions, the entirety of the displacement throughout all of 3D space can be captured in 4D space by looking at only one period of the AMF as shown in the zoomed-in region. Reproduced with permission of the International Union of Crystallography.

from one state to the next. This information can also be used to generate animations of how the atoms are modulating in superspace.

6.4. Case studies

6.4.1. Profilin:actin (PA) complex

Incommensurately modulated crystals of the PA complex have been known since the 1980s [31]. It was not until the early 2000s that software became available to investigate these samples more completely. Figure 30 shows an incommensurately modulated diffraction pattern obtain from a PA crystal. The main reflections have been highlighted. Satellite reflections are visible flanking each main on either side. Only first-order satellites are visible. Although it is not possible to determine the shape of the modulation function from the intensities of the satellite reflections, it is possible to infer that the function is very smooth with no sharp changes because if there were sharp changes then higher-order satellites (more than one) would be visible on either side of the main reflection.

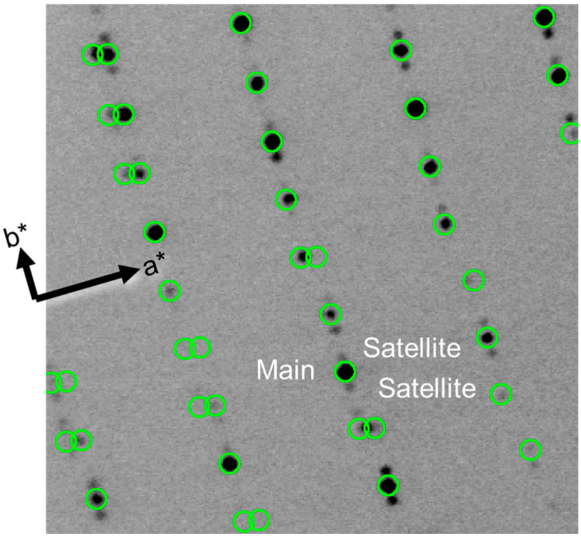

Figure 30. Incommensurately modulated diffraction pattern from a profilin:actin crystal shown with mains highlighted by circles and satellites on either side of the mains (not circled) for most of the reflections.

Twinsolve [48] was originally used to index first data but this software was unable to integrate the reflections [71]. Then, several years later, Eval15 [50] was used to integrate and index the reflections from three datasets. The publication by Porta and colleagues [49] has a flow diagram on how to process this type of data with Eval15. The superspace group was found to be $P2_12_12_1(0\beta0)$ with $x_1 = 37.1$ Å, $x_2 = 71.1$ Å, $x_3 = 185.1$ Å and $\mathbf{q} = 0\mathbf{a}^* + 0.2829\mathbf{b}^* + 0\mathbf{c}^*$. Using currently available software tools the only way to solve this structure is to use a commensurate approximation. For PA, the closest commensurate approximation is a 7x supercell which contains 2 modulation waves. Additionally, the reflections need to be reindexed into 3D space. The reindexing is dependent on the supercell. The first step is to refine the average structure. There are two options that can be used to determine the average structure. The first is to use only the main reflections and perform molecular replacement. The other option is to add the satellite reflection intensities to the associated main reflections and use these new reflections for molecular replacement. In the case of PA, there is no difference between either approach based on the molecular replacement statistics. The average structure is expected to have poor electron density around parts of the structure that have the highest amount of modulation (Figure 31). Actin subdomains A2 and A4 (Figure 31(a)) have the weakest electron density. This makes sense because by analyzing the PA structure, actin is shaped like Pac-Man with A2 and A4 forming the mouth that can open and close (Figure 31(b)).

Once the average structure has been fit, it can be used to refine all the reflection data by expanding the number of average structures to fill a supercell. Another option, at this point, would be to search for multiple copies of the average structure in the supercell using molecular replacement with all the reflections. Refinement of the supercell structure of PA is ongoing toward a publishable refinement. In this case, a satisfactory result would be relatively low R/R_{free} values, good electron density throughout the supercell model, and modulation functions that are smoothly varying for the atoms. Fourier and real superspace

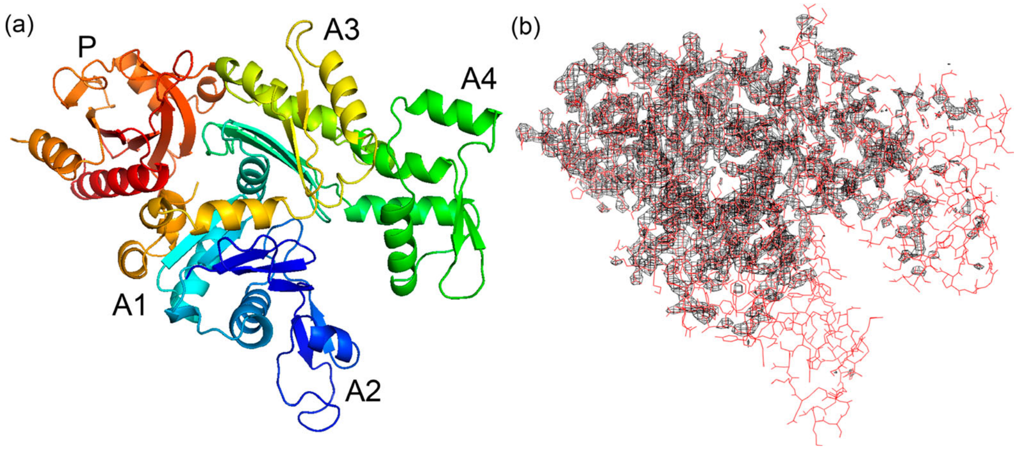

Figure 31. Profilin:Actin complex. (a) Cartoon representation of PA with profilin labelled P and actin labelled with its subdomains A1–A4. (b) Unpublished profilin:actin average structure refinement with the orientation shown the same as in part a showing poor density ($2F_o$–F_c at 2.0 sigma) for the model in regions A2 and A4 of actin.

constraints are being tested that will hopefully help guide the refinement and produce higher quality models with respect to the data.

The initial supercell approximation refinement of modulated profilin:actin appears to be progressing in the right direction. As stated earlier, with only one satellite reflection visible, the function describing the atomic displacement in superspace should be smooth, approaching a sine wave. Superspace displacement data can be represented on a t-plot (Incommensurate Crystallography section 2.4 [90]) where the value t represents the phase of the atomic modulation function where integer values represent whole periods of the function. The atomic displacements of profilin and actin can be plotted as a function of the displacement of their centre-of-mass in superspace (Figure 32). The t-plot of the centre-of-mass shows globally how the molecules are behaving in superspace. First, the displacement seems to be constrained to the $\boldsymbol{x}$ and $\boldsymbol{y}$ directions. Second, the displacement is smooth as expected. Third, actin is modulating the most and moves the most in the $\boldsymbol{y}$-direction. Finally, actin and profilin move about the same amount in the **z**-direction. Because of how superspace operates molecules next to each other in real space are spaced further apart from each other (about 0.1 t) in superspace (Figure 32 two black vertical dashed lines). This leads to the conclusion that profilin and actin move away from each other in $\boldsymbol{y}$ and move more or less in sync with one another in **z** with the profilin slightly leading the actin in displacement.

6.4.2. *Hyp-1-ANS complex*

The Hyp-1-ANS complex was discussed previously [56] in section 5.3.1 as a tNCS case study. Diffraction problems that can be solved using a supercell sit at a unique crossroads where they can be both described using the superspace approach or the supercell approach [52]. The diffraction pattern for Hyp-1-ANS can be interpreted as a (3 + 1)D modulation where the **q** vector is rational and so the modulation is commensurate leading to the original solution as a supercell. The difficulty with a modulated diffraction is in assigning the

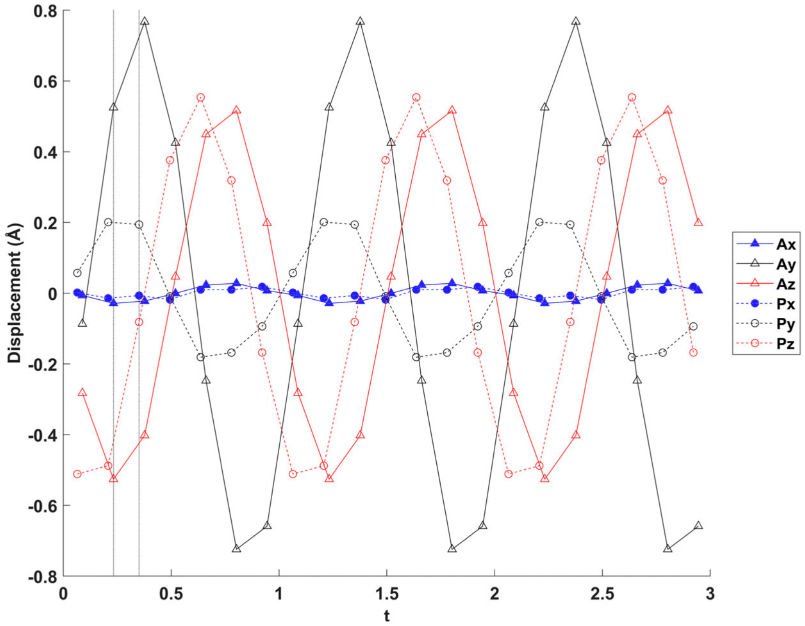

Figure 32. Centre of mass displacements for Profilin and Actin in superspace.

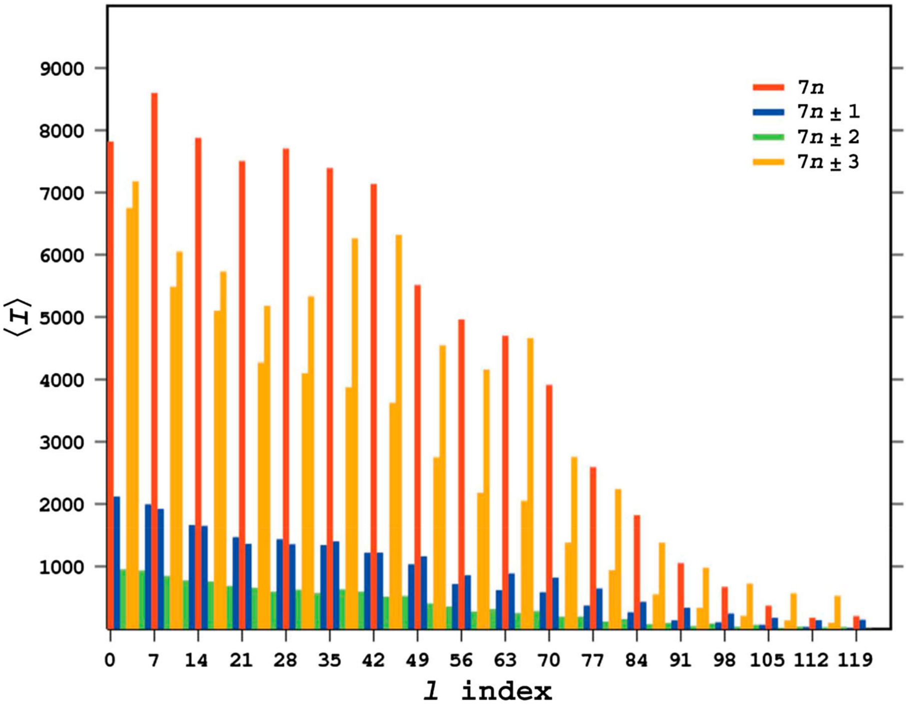

Figure 33. Hyp-1-ANS reflection intensity plot as a function of l index [55]. Reproduced with permission of the International Union of Crystallography.

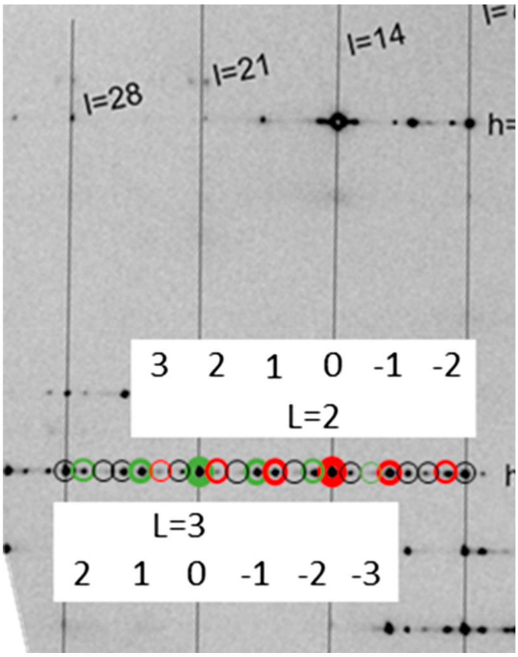

Figure 34. Superspace indexing for two of the main reflections. The first one shows the main (L = 3 where in the supercell it was L = 21) and lower order satellites highlighted with green circles. The width of the circle is related to the order of the reflection (thickest main to thinnest 3rd order satellite). The second (L = 2) is shown in red. Reflections with black circles represent satellites or mains that do not belong to either L = 2 or L = 3 main reflections (Adapted from [55]). Reproduced with permission of the International Union of Crystallography.

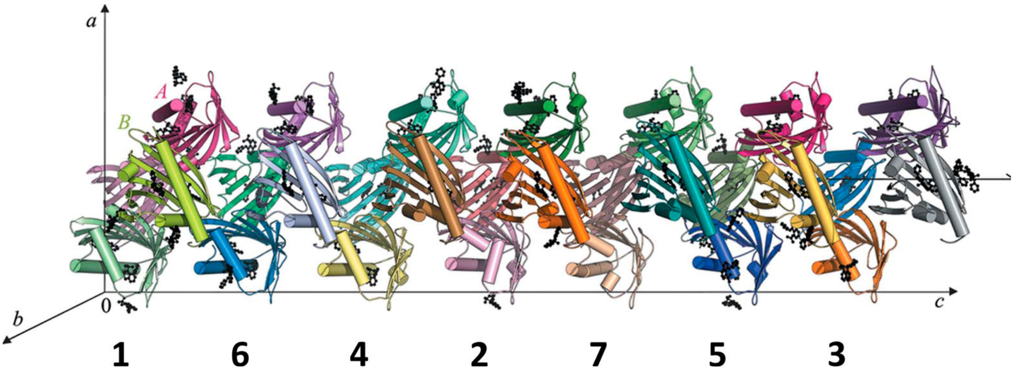

Figure 35. Required reordering of cells to view the atomic displacements in superspace for a 3/7 commensurate modulation (Adapted from [55]). Reproduced with permission of the International Union of Crystallography.

proper indexing to the **q** vector. In this case, the assignment becomes much easier because the authors provided a nice analysis of the reflection intensities as a function of *l* (the direction where the modulation has occurred) in another publication [55]. The main reflections can be assigned as the ones that have on average the strongest intensities. Satellites have

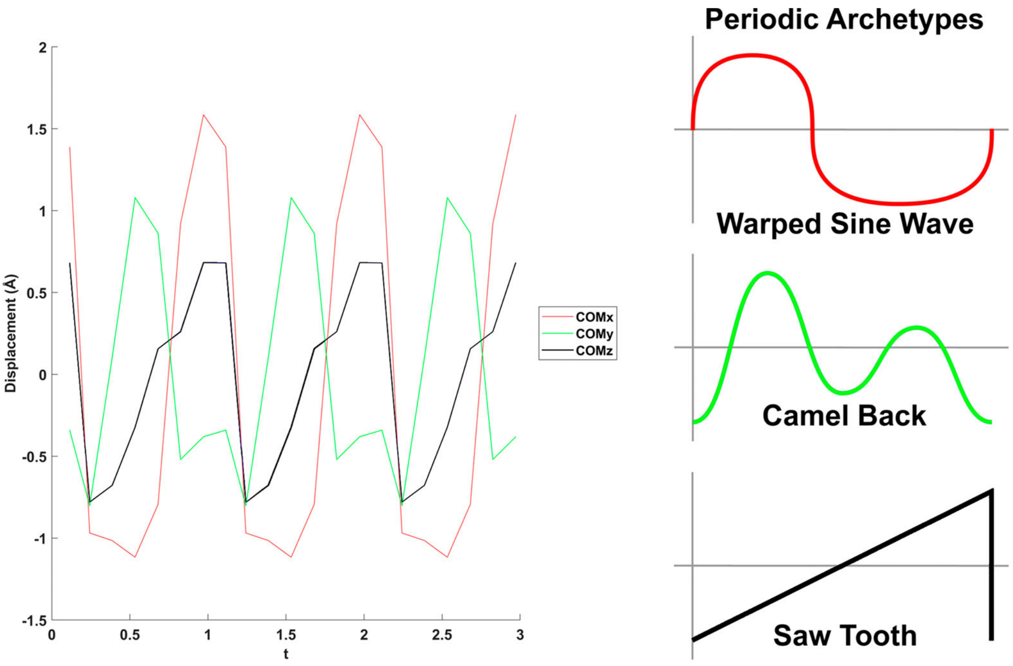

Figure 36. Hyp-1-ANS centre of mass displacements in superspace showing three periods of the modulation functions and the associated archetype functions to the right.

weaker intensities on average than the main. Looking over the intensities (Figure 33), that the main reflections are the most intense followed by the first order satellites who are followed by the second order satelites and finally the third order satellites (weakest on average) and that the main reflections are spaced every seven. So mains have a value of $7n$ and first order satellites are the ones with $7n \pm 3$ and second order satellites are $7n \pm 1$, and finally, third order are $7n \pm 2$ which leads to a **q** vector with a single component along $\mathbf{l}^*$ or h_3 with a **q** vector of $0\mathbf{a}^* + 0\mathbf{b}^* + 3/7\mathbf{c}^*$.

All of the reflections can be described with a single **q** vector which makes this a $(3+1)$D modulation which means the reflections can be indexed with four indices. Figure 34 shows how the reflection indexing in superspace would look. In this case, the satellites are dispersed among one another to the point where the third-order satellite has a main between itself and the main to which it belongs. With several orders of satellite reflections measurable for each main, the modulation functions would also be expected to more complex than simple sine waves. An estimate of the shape of the modulation function can be constructed by reorganizing the supercell structure (Figure 35) and plotting the positions of the centre of mass of the chains in superspace (Figure 36).

The resulting plots (Figure 36) shows some interesting features for the Hyp-1-ANS modulation functions. Along the $\boldsymbol{x}$-direction, the periodic function appears to be a warped sine wave were the peaks are compressed and the valleys are elongated. In the $\boldsymbol{y}$-direction, the periodic function is similar to a camel back with two humps. In the **z**-direction, there function resembles a sawtooth pattern where the modulation slowly grows to some maximum and then almost instantaneous resets itself back at a minimum to repeat the slow

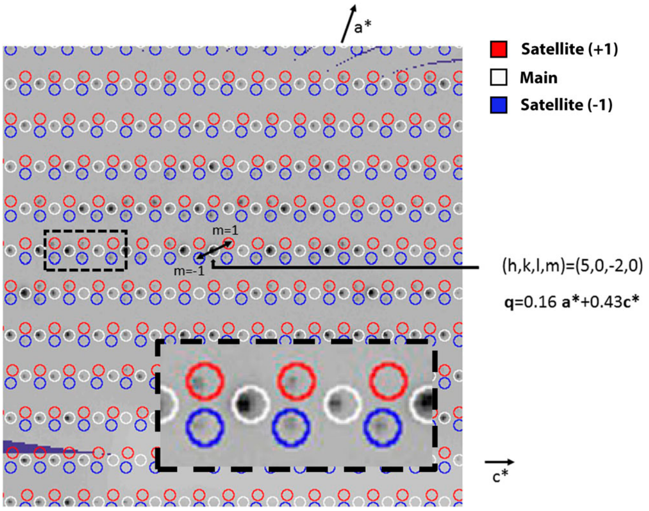

Figure 37. NAL indexed incommensurately modulated diffraction image created with Precession in EVAL15 (Main reflections and satellites are highlighted with circles) [54].

increase. These more complicated functions as compared to the PA example were expected because of the higher order satellites that were observed.

6.4.3. N-acetyl-neuraminic lyase (NAL)

Several crystals of NAL have been found that have incommensurately modulated diffraction patterns [54]. Several different forms have been crystallized. Four forms identified by Campeotto and coworkers were incommensurately modulated with space group and average unit cell parameters of $P2_1$ with $a = 54.8$ Å, $b = 142.2$ Å, $c = 84.2$ Å, $\alpha = 90°$ $\beta = 108.97°$ $\gamma = 90°$, and $\mathbf{q} = 0.16\mathbf{a}^* + 0.0\mathbf{b}^* + 0.43\mathbf{c}^*$. NAL indexing is the first report of a **q** vector with a direction that is not aligned along the crystallographic axis for a macromolecule. The incommensurately modulated data were processed with Eval15 (Figure 37). Strong, but only first-order satellites, are visible. This would indicate that the modulation is smooth. Additionally, these crystals suffered from twinning. Although not clearly stated by the authors, apparently only the main reflections were used for structure solution and refinement. They were able to get satisfactory results for all their modulated crystals but one. For this example, it would be interesting to see how a supercell approximation refinement would work using the **q** vector to determine the appropriate supercell.

7. Conclusions

In summary, four classes of difficult pathological crystals were discussed in detail by analysing examples from literature. Two of the classes are derived from order–disorder: ROD and LTD. The other two are related to higher-order periodic modulation in the sample: tNCS and Modulated Crystals. An easy to follow, experimental pathway to solve each malady is provided in Table 2 where each defect class, the symptoms, and how to solve the structure are summarized. The defects are listed from easiest to solve (ROD) to the

Table 2. The four main categories of pathological crystals analyzed for this review. Subjective.

Crystal Issue	Description	Symptoms	Solution
Rotational Order/Disorder (ROD)<br>Section 3	In the crystal components are incorporated with a rotation relative to the other components randomly throughout the sample	• Streaks in the diffraction pattern<br>• Poor density in large regions of the map<br>• I422 space groups<br>• Un-modelled overlapping ghost density	• Make sure that the sample is not twinned<br>• Make sure the sample is not merahedrally twinned<br>• Model in the rotated overlapping component and refine for occupancy<br>• Solve as P1
Layer Translocation Defect (LTD)<br>Section 4	Well-formed layers but some layers are randomly shifted with respect to a most favoured arrangement	• Streaky diffraction along specific directions<br>• Poor electron density layers in the structure	• Make sure that the sample is not twinned<br>• Correct the reflection intensities to remove the translocation effect and then solve the resulting density
Translational Non-Crystallographic Symmetry (tNCS)<br>Section 5	Occurs in crystals where there are multiple copies ($\gg$2) of a chain in the asymmetric unit cell that are slightly shifted/rotated with respect to one another	• Observed by analysing the Patterson map and finding strong peaks that are not supposed to exist for a given space group<br>• Problems when trying to perform molecular replacement	• Make sure that the sample is not twinned<br>• Can be handled by existing software<br>• Depending on the number of tNCS operators the software may need help properly locking into the solution
Modulation<br>Section 6	The crystal has a periodic long-range order that may or may not be commensurate with the unit cell	• Unit cell main reflections will be flanked by one or more satellite reflections that in general will be of decreasing intensity as function of distance from the main	• Make sure that the sample is not twinned<br>• If it is commensurate a supercell may be used to process the data<br>• If it is incommensurate then a supercell approximation may be used to get a rough look at the solution<br>• Data can be indexed and integrated with Eval15

most difficult (incommensurately modulated). In many cases, there are early indicators of a problem. For example, if the diffraction pattern has regions of sharp and diffuse reflections then ROD or LTD are indicated. If the processing statistics are not as good as would be expected, expect trouble. If all the reflections cannot be indexed with a single matrix or multiple matrices (in the case of twinning) then you might have an incommensurate modulation. Problems can also appear after data processing. For example, the Mathews coefficient can return a confusing value with ROD. Molecular replacement might initially fail to find a good solution if there is tNCS or a modulation of some kind. The molecular replacement solutions might have large areas of intersecting unfit density (ghost density) that looks like areas of the structure that are in density. The intensity distribution in the reflections could show a modulated pattern. The native Patterson map could have unexplained large off origin peaks. These symptoms all indicate you have a pathological crystal to solve.

In the end, it usually takes a detail-oriented person who refuses to give up to solve the structure. Fortunately, the number and quality of software tools to help solve these problems have been improving. Additionally, awareness in the scientific community is better and collaborative help is more readily available. Progress will continue along multiple fronts, and these types of issues will be supported and handled smoothly by the software programmes in the future.

This review has shown that although some datasets can be challenging that with enough perseverance and creative problem solving they can be overcome and solved. We would encourage the community to collect full datasets on these types of samples even so that they can be used by other researchers to improve software programmes or make additional tools available to researchers. More so at synchrotron sources where the detectors and beam intensity allow for a full 180-degree data collection very quickly. It would be interesting to use a technique like CryoEM to actually visualize this disorder at the molecular level. It may also be interesting to use a technique like XFEL to see if these same sorts of issues are visible in very small crystalline samples or if these regions of disorder occur in larger domains only.

Acknowledgments

We collaborated with Jason Porta, Clarence Schutt, Uno Lindberg, Kartik Natarajan on these topics over the years. We thank Vaclav Petricek, Garib Murshudov, Sander van Smaalen, Lukas Palatinus, Joe Ferrara, Christer Svensson, Toine Schreurs and Loes Kroon-Batenburg for useful discussion. We thank the University of Nebraska Medical for their support and in particular the Eppley Institute. GB acknowledges the following funding agencies NASA, NSF, Nebraska Research Initiative, NIH, NCI. We also thank all the researchers who persevered and solved their pathological crystal structures.

Disclosure statement

No potential conflict of interest was reported by the authors.

Funding

This work was supported by National Science Foundation [grant number 1518145].

Notes on contributors

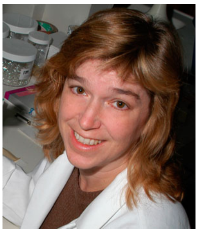

Gloria E. O. Borgstahl, born the youngest of seven in Dubuque, Iowa, is a Professor in the Eppley Institute for Research in Cancer and Allied Diseases at the University of Nebraska Medical Center (UNMC) in Omaha, Nebraska in the USA. She earned her Bachelor in Science and Engineering degree (B.S.E.) and her Ph.D. in Biochemistry from the University of Iowa in Iowa City, Iowa in 1985 and 1992, respectively. After postdoctoral experiences with Dr. Elizabeth Getzoff and Dr. John Tainer at the Scripps Research Institute in La Jolla, CA and with Dr. Thomas Terwilliger at Los Alamos National Laboratory in Los Alamos, NM, she became an Assistant Professor of Chemistry at the University of Toledo in Toledo, OH. Her research interests in cancer and the structural biology of DNA double-strand break repair brought her to UNMC in 2002. She has regularly attended the Gordon Conference on Diffraction Methods, the IUCr meetings and has been somewhat helpful in the past to the General Interest Group of the ACA. She is currently a member of the Aperiodic Commission for the IUCr. Her main hobbies include anything her children want to do which over the years has included golf, long distance bike riding (RAGBRAI and BRAN in particular), intensely listening and studying music (Led Zepplin, Green Day, Frank Zappa, Red Hot Chili Peppers, and etc.) and its history, Frisbee, our many types of pets and going to international meetings and pretending we live there.

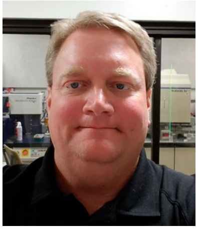

Jeffrey J. Lovelace is the x-ray laboratory manager at the Eppley Institute for Research in Cancer and Allied Diseases at the University of Nebraska Medical Center (UNMC) in Omaha, Nebraska in the USA. He received a Bachelor of Science in Engineering Physics from the University of Toledo in 1995. After a brief stint at a small engineering firm prototyping and deploying high-speed inspection equipment for use in various industries, he went back to the University of Toledo on to earn a Master of Science in Bioengineering focusing on Artificial Intelligence in 1998. Then, through a collection of adventures, ended up doing protein crystallography for the purpose of measuring the quality of microgravity grown crystal samples and finally into incommensurate protein crystallography in Gloria Borgstahl's laboratory at the University of Toledo and at the Eppley Institute in Omaha, NE. He regularly attends the ACA and IUCr meetings as well as the occasional Gordon Conference. His hobbies include golf, snow skiing, shooting trap and skeet, beer and wine tasting but never IPAs.

ORCID

Jeffrey J. Lovelace http://orcid.org/0000-0002-4217-8371
Gloria E. O. Borgstahl http://orcid.org/0000-0001-8070-0258

References

[1] Helliwell JR. Macromolecular crystal twinning, lattice disorders and multiple crystals. Cryst Rev. 2008;14(3):189–250.
[2] Parsons S. Introduction to twinning. Acta Cryst. 2003;D59:1995–2003.
[3] Yeates TO. Detecting and overcoming crystal twinning. Method Enzymol. 1997;276:344–358.
[4] Yeates TO, Fam BC. Protein crystals and their evil twins. Structure. 1999;7(2):R25–R29.
[5] Roversi P, Blanc E, Johnson S, Lea SM. Tetartohedral twinning could happen to you too. Acta Cryst. 2012;D68:418–424.
[6] Patterson BD. Crystallography using an X-ray free-electron laser. Cryst Rev. 2014;20(4):242–294.
[7] Sauter, NK. XFEL diffraction: developing processing methods to optimize data quality. J Synchrotron Radiat. 2015;22:239–248.
[8] Christensen KE, Thompson AL. Improving our understanding of modulation in molecular materials. Ames (IA), 2018.
[9] Durbin SD, Feher, G. Protein crystallization. Annu Rev. 1996;47(1):171–204.

[10] Li XX, Xu XD, Dan YY, Zhang ML. The factors during protein crystallization: a review. Cryst Reports. 2008;53(7):1261–1266.
[11] McPherson A, Gavira JA. Introduction to protein crystallization. Acta Cryst F. 2013;70(Pt 1):2–20.
[12] Nanev CN. Recent experimental and theoretical studies on protein crystallization. Cryst Res and Tech. 2017;52(1):1600210.
[13] Zhou RB, Cao HL, Zhang CY, Yin CY. A review on recent advances for nucleants and nucleation in protein crystallization. Cryst Eng Comm. 2017;19(8):1143–1155.
[14] Howells ER; Perutz MF. The structure of Haemoglobin – V. Imidazole-Methaemoglobin: a further check of the signs. Proc R Soc London A Math Phys Sci. 1954;225(1162):308–314.
[15] Cochran W, Howells ER. X-ray diffraction by a layer structure containing random displacements. Acta Cryst. 1954;7:412–415.
[16] Bragg WL, Howells ER. X-ray diffraction by imidazole methaemoglobin. Acta Cryst. 1954;7:409–411.
[17] Dornberger-Schiff K. On order-disorder structures (OD-structures). Acta Cryst. 1956;9(7):593–601.
[18] Dornberger-Schiff K, Grell-Niemann H. On the theory of order-disorder (OD) structures. Acta Cryst. 1961;14(2):167–177.
[19] Glauser S, Rossmann MG. Disorder in erythrocyte catalase crystals. Acta Cryst. 1966;21(1):175–177.
[20] de Wolff PM. Symmetry operations for displacively modulated structures. Acta Cryst A. 1977;33(3):493–497.
[21] Janner A, Janssen T. Symmetry of periodically distorted crystals. Phys Rev B. 1977;15(2):643–658.
[22] Janner A, Janssen T. Symmetry of incommensurate crystal phases. II. Incommensurate basic structure. Acta Cryst A. 1980;36(3):408–415.
[23] Janner A, Janssen T. Symmetry of incommensurate crystal phases. I. Commensurate basic structures. Acta Cryst A. 1980;36(3):399–408.
[24] de Wolff PM, Janssen T, Janner A. The superspace groups for incommensurate crystal structures with a one-dimensional modulation. Acta Cryst A. 1981;37(5):625–636.
[25] Fourier JB. Theorie analytique de la chaleur [microform] / parM. Fourier. Paris: F. Didot, 1822.
[26] Burkhard H. Trigonometrische Interpolation. Encyklopddie der Mathematischen Wissenschaften. 1899–1916.
[27] Cooley J, Tukey J. An algorithm for the machine calculation of complex Fourier series. Math Comp. 1965;19(90):297–301.
[28] Frigo M, Johnson SG. The Design and Implementation of FFTW3. Proc of the IEEE. 2005;93(2): 216–231.
[29] Pickersgill R. One-dimensional disorder in spinach ribulose bisphosphate carboxylase crystals. Acta Cryst A. 1987;43(4):502–506.
[30] Brick P, Bhat TN, Blow DM. Structure of Tyrosyl-tRNA synthetase refined at 2.3A resolution. J Mol Biol. 1989;208:83–98.
[31] Schutt CE, Lindberg U, Myslik J, Strauss N. Molecular packing in profilin: actin crystals and its implications. J Mol Biol. 1989;209(4):735–746.
[32] Schulze-Gahmen U, Rini JM, Wilson IA. Detailed analysis of the free and bound conformations of an antibody: X-ray structures of fab 17/9 and three different fab-peptide complexes. J Mol Biol. 1993;234(4):1098–1118.
[33] Lietzke SE, Carperos VE, Kundrot CE. Data reduction from twinned RNA crystals. Acta Cryst D. 1996;52(4):687–692.
[34] Dauter Z, Botos I, LaRonde-LeBlanc N, Wlodawer A. Pathological crystallography: case studies of several unusual macromolecular crystals. Acta Cryst D. 2005;61(7):967–975.
[35] Merritt EA, Sarfaty S, Feil IK, Hol WG. Structural foundation for the design of receptor antagonists targeting Escherichi coli heat-labile enterotoxin. Structure. 1997;5(11):1485–1499.
[36] Kamtekar S, Berman AJ, Wang J, Lázaro JM, de Vega M, Blanco L, Salas M, Steitz TA. Insights into strand displacement and processivity from the crystal structure of the protein-primed DNA polymerase of bacteriophage ϕ29. Mol Cell. 2004;16(4):609–618.
[37] Patterson AL. A direct method for the determination of the components of interatomic distances in crystals. Z Kristallogr Cryst Mater. 1935;90(1–6):517–542.

[38] Wang J, Kamtekar S, Berman AJ, Steitz, TA. Correction of X-ray intensities from single crystals containing lattice-translocation defects. Acta Cryst. 2005;D61:67–74.
[39] Wang J, Rho SH, Park HH, Eom SH. Correction of X-ray intensities from an HsIV-HsIU co-crystal containing lattice-translocation defects. Acta Cryst. 2005;D61:932–941.
[40] Hwang WC, Lin Y, Santelli E, Sui J, Jaroszewski L, Stec B, Farzan M, Marasco WA, Liddington RC. Structural basis of neutralization by a human anti-severe acute respiratory syndrome spike protein antibody, 80R. J Biol Chem. 2006;281(45):34610–34616.
[41] Rye CA, Isupov MN, Lebedev AA, Littlechild JA. An order–disorder twin crystal of L-2-haloacid dehalogenase from Sulfolobus tokodaii. Acta Cryst. 2007;D63:926–930.
[42] Dhillon AK, Stanfield RL, Gorny MK, Williams C, Zolla-Pazner S, Wilson IA. Structure determination of an anti-HIV-1 Fab 447-52D-peptide complex from an epitaxially twinned data set. Acta Cryst D. 2008;64(7):792–802.
[43] Zhu X, Xu X, Wilson IA. Structure determination of the 1918 H1N1 neuraminidase from a crystal with lattice-translocation defects. Acta Cryst. 2008;D64:843–850.
[44] Hare S, Cherepanov P, Wang J. Application of general formulas for the correction of a lattice-translocation defect in crystals of a lentiviral integrase in complex with LEDGF. Acta Cryst. 2009;D65: 966–973.
[45] Pletnev S, Morozova KS, Verkhusha VV, Dauter Z. Rotational order-disorder structure of fluorescents protein FP480. Acta Cryst. 2009;D65:906–912.
[46] Pletnev S, Subach FV, Verkhusha VV, Dauter Z. The rotational order–disorder structure of the reversibly photoswitchable red fluorescent protein rsTagRFP. Acta Cryst. 2014;D70:31–39.
[47] Renko M, Taler-Vercic A, Mihelic M, Žerovnik E, Turk D. Partial rotational lattice order-disorder in stefin B crystals. Acta Cryst. 2014;D70:1015–1025.
[48] [48] Svensson C. TwinSolve. 2003.
[49] Porta J, Lovelace JJ, Schreurs AMM, Kroon-Batenburg LMJ, Borgstahl GEO. Processing incommensurately modulated protein diffraction data with Eval15. Acta Cryst. 2011;D67(Pt 7):628–638.
[50] Schreurs AMM, Xian X, Kroon-Batenburg LMJ. EVAL15: a diffraction data integration method based on ab initio predicted profiles. J Appl Cryst. 2010;43:70–82.
[51] Lovelace JJ, Winn MD, Borgstahl GEO. Simulation of modulated reflections. J Appl Cryst. 2010;43(2):285–292.
[52] Lovelace JJ, Simone PD, Petříček V, Borgstahl GEO. Simulation of modulated protein crystal structure and diffraction data in a supercell and in superspace. Acta Cryst D. 2013;69(6):1062–1072.
[53] Porta J, Lovelace J, Borgstahl GEO. How to assign a (3 + 1)-dimensional superspace group to an incommensurately modulated biological macromolecular crystal. J Appl Cryst. 2017;50(4):1200–1207.
[54] Campeotto I, Lebedev A, Schreurs AMM, Kroon-Batenburg LMJ, Lowe E, Phillips SEV, Murshudov GN, Pearson AR. Pathological macromolecular crystallographic data affected by twinning, partial-disorder and exhibiting multiple lattices for testing of data processing and refinement tools. Sci Rep. 2018;8(1):14876.
[55] Sliwiak J, Dauter Z, Kowiel M, McCoy AJ, Read RJ, Jaskolski M. ANS complex of St John's wort PR-10 protein with 28 copies in the asymmetric unit: a fiendish combination of pseudosymmetry with tetartohedral twinning. Acta Cryst D. 2015;71(Pt 4):829–843.
[56] Sliwiak J, Jaskolski M, Dauter Z, McCoy AJ, Read RJ. Likelihood-based molecular-replacement solution for a highly pathological crystal with tetartohedral twinning and sevenfold translational noncrystallographic symmetry. Acta Cryst D. 2014;70(Pt 2):471–480.
[57] McCoy AJ, Grosse-Kunstleve RW, Adams PD, Winn MD, Storoni LC, Read RJ, Phaser crystallographic software. J Appl Cryst. 2007;40(4):658–674.
[58] Read RJ; Adams PD, McCoy AJ. Intensity statistics in the presence of translational noncrystallographic symmetry. Acta Cryst D. 2013;69(2):76–183.
[59] Sundlov JA, Gulick AM. Structure determination of the functional domain interaction of a chimeric nonribosomal peptide synthetase from a challenging crystal with noncrystallographic translational symmetry. Acta Cryst D. 2013;69(8):1482-1492.

[60] Donovan KA, Atkinson SC, Kessans SA, Peng F, Cooper TF, Griffin MD, Jameson GB, Dobson RC. Grappling with anisotropic data, pseudomerohedral twinning and pseudo-translational noncrystallographic symmetry: a case study involving pyruvate kinase. Acta Cryst D. 2016;72(4):512–519.
[61] Read RJ, McCoy AJ. A log-likelihood-gain intensity target for crystallographic phasing that accounts for experimental error. Acta Cryst D. 2016;72(3):375–387.
[62] Reimer JM, Aloise MN, Powell HR, Schmeing TM. Manipulation of an existing crystal form unexpectedly results in interwoven packing networks with pseudo-translational symmetry. Acta Cryst D. 2016;72(10):1130–1136.
[63] Sporny M, Guez-Haddad J, Waterman DG, Isupov MN, Opatowsky Y. Molecular symmetry-constrained systematic search approach to structure solution of the coiled-coil SRGAP2 F-BARx domain. Acta Cryst D. 2016;72(12):1241–1253.
[64] Sheldrick GM. XPREP. Madison; 2008.
[65] Sheldrick GM. Crystal structure refinement with SHELXL. Acta Cryst C. 2015;71(1):3–8.
[66] French S, Wilson K. On the treatment of negative intensity observations. Acta Cryst A. 1978;34(4):517–525.
[67] Winn MD, Ballard CC, Cowtan KD, Dodson EJ, Emsley P, Evans PR, Keegan RM, Krissinel EB, Leslie AG, McCoy A, McNicholas SJ, Murshudov GN, Pannu NS, Potterton EA, Powell HR, Read RJ, Vagin A, Wilson KS. Overview of the CCP4 suite and current developments. Acta Cryst D. 2011;67(4):235–242.
[68] Padilla JE, Yeates TO. A statistic for local intensity differences: robustness to anisotropy and pseudo-centering and utility for detecting twinning. Acta Cryst D. 2003;59(7):1124–1130.
[69] Zwart P, Grosse-Kunstleve RW, Adams P. Xtriage and Fest: automatic assessment of X-ray data and substructure structure factor estimation. CCP4 Newsletter. 2005 01/01, 43.
[70] Adams PD, Afonine PV, Bunkoczi G, Chen VB, Davis IW, Echols N, Headd JJ, Hung LW, Kapral GJ, Grosse-Kunstleve RW, McCoy AJ, Moriarty NW, Oeffner R, Read RJ, Richardson DC, Richardson JS, Terwilliger TC, Zwart PH. PHENIX: a comprehensive Python-based system for macromolecular structure solution. Acta Cryst D. 2010;66(2):213–221.
[71] Lovelace JJ, Murphy CR, Daniels L, Narayan K, Schutt CE, Lindberg U, Svensson C, Borgstahl GEOB. Protein crystals can be incommensurately modulated. J Appl Cryst. 2008;41:600–605.
[72] Winter G, Waterman DG, Parkhurst JM, Brewster AS, Gildea RJ, Gerstel M, Fuentes-Montero L, Vollmar M, Michels-Clark T, Young ID, Sauter NK, Evans G. DIALS: implementation and evaluation of a new integration package. Acta Cryst D. 2018;74(2):85–97.
[73] Vagin A, Teplyakov A. MOLREP: an Automated Program for Molecular Replacement. J Appl Cryst. 1997;30(6):1022-1025.
[74] Petříček V, Dušek M, Palatinus L. Crystallographic computing system JANA2006: general features. Z Kristallogr Cryst Mater. 2014;229(5):345–352.
[75] Murshudov GN, Skubak P, Lebedev AA, Pannu, NS, Steiner RA, Nicholls RA, Winn MD, Long F, Vagin AA. REFMAC5 for the refinement of macromolecular crystal structures. Acta Cryst D. 2011;67(4):355–367.
[76] Afonine PV, Grosse-Kunstleve RW, Echols N, Headd JJ, Moriarty NW, Mustyakimov M, Terwilliger TC, Urzhumtsev A, Zwart PH, Adams PD. Towards automated crystallographic structure refinement with phenix.refine. Acta Cryst D. 2012;68(4):352–367.
[77] Turk D. MAIN software for density averaging, model building, structure refinement and validation. Acta Cryst D. 2013;69(8):1342-1357.
[78] Palatinus L, Chapuis G. SUPERFLIP - a computer program for the solution of crystal structures by charge flipping in arbitrary dimensions. J Appl Cryst. 2007;40(4):786–790.
[79] Emsley P, Lohkamp B, Scott WG, Cowtan K. Features and development of Coot. Acta Cryst D. 2010;66(4):486–501.
[80] Schrodinger LLC. The PyMOL molecular graphics System, Version 1.8. 2015.
[81] The Mathworks Inc. Matlab. 2007b. The Mathworks Inc.; 2007.
[82] RStudio Team. RStudio: integrated development for R. Boston, MA: RStudio, Inc.; 2016.
[83] R Core Team. R: a language and environment for statistical computing. Vienna, Austria; 2017.

[84] Lovelace JJ, Murshudov G, Petříček V, Borgstahl GEOB. Hyper-restraints: improving super-cell approximation refinements of an incommensurately modulated protein. Acta Cryst A. 2018;74(a1): a229.
[85] Weichenberger CX, Rupp B. Ten years of probabilistic estimates of biocrystal solvent content: new insights via nonparametric kernel density estimate. Acta Cryst D. 2014;70(6):1579–1588.
[86] Matthews BW. Solvent content of protein crystals. J Mol Biol. 1968;33(2):491–497.
[87] Kantardjieff KA, Rupp B. Matthews coefficient probabilities: improved estimates for unit cell contents of proteins, DNA, and protein-nucleic acid complex crystals. Protein Sci. 2003;12(9):1865–1871.
[88] Ponnusamy R, Lebedev AA, Pahlow S, Lohkamp B. Crystal structure of human CRMP-4: correction of intensities for lattice-translocation disorder. Acta Cryst. 2014;D70:1680–1694.
[89] Ruf A, Tetaz T, Schott B, Joseph C, Rudolph MG. Quadruple space-group ambiguity owing to rotational and translational noncrystallographic symmetry in human liver fructose-1,6-bisphosphatase. Acta Cryst D. 2016;72(11):1212–1224.
[90] van Smaalen, S. Incommensurate crystallography. Oxford: Oxford University Press; 2007.

Index

Authors
- Campeotto. *See* NAL
- Dauter. *See* rsTagRFP
- Jaskolski. *See* Hyp-1-ANS
- Lohkamp. *See* CRMP-4
- Pearson. *See* NAL
- Pletnev. *See* rsTagRFP
- Read. *See* Hyp-1-ANS
- Rudolph. *See* FBPase
- Turk. *See* stefin B
- Wang. *See* LEDGF

Bragg, 6, 8, 9
commensurate, 34
Coot. *See* Software
CRMP-4, 25, 26
CryoEM, 43
DIALS. *See* Software
Eval15. *See* Software
FBPase, 29, 30
Hyp-1-ANS, 28, 37
incommensurate, 5, 8, 9, 12, 32, 33, 34, 47
incommensurate diffraction, 33
incommensurate modulation, 5
Jana2006. *See* Software, See Software
layer translocation defect. *See* LTD
LEDGF, 21, 22
LTD, 7, 8, 9, 21, 22, 23, 24, 25, 27, 41, 43
MAIN. *See* Software
Matlab. *See* Software
Matthew's Coefficient, 15

modulated structures, 5, 12, 33
MOLREP. *See* Software
NAL, 41
Order–Disorder, 7, 8, 14, *See* LTD, *See* ROD
PA, 8, 10, 35, 36, 41
Patterson map, 9, 10, 21, 23, 27, 29, 30, 43
Perutz, 6
Phaser. *See* Software
Phenix.refine. *See* Software
Phenix.xtriage. *See* Software
Profilin:Actin Complex. *See* PA
PyMOL. *See* Software
q-vector, 33, 34, 37, 39, 40, 41
REFMAC5. *See* Software
ROD, 9, 14, 15, 18, 41, 43
rotational order–disorder, 5, 9, *See* ROD
rsTagRFP, 9, 15
RStudio. *See* Software
Satellite reflections, 8, 9, 33, 34, 35, 40, 42
Software
  Coot, 13, 17, 18
  DIALS, 12
  Eval15, 10, 11, 12, 34, 36, 41, 42
  Jana2006, 12, 33
  MAIN, 13, 18
  Matlab, 14
  MOLREP, 12, 16, 17
  Phaser, 10, 12, 18, 28, 29
  Phenix.refine, 13, 17
  Phenix.xtriage, 11, 15, 18
  PyMOL, 14
  REFMAC5, 13, 14, 17, 18, 26
  RStudio, 14
  Superflip 13, 33 *See* software
  TRUNCATE, 11, 15
  Twinsolve, 10, 11, 36
  UCLA Twin Detection Server, 11, 15
  XPREP, 11, 15
stefin 9, 18, 19, 20
supercell, 10, 32, 34, 36, 37, 39, 40, 41, 42
supercell approximation, 34, 37, 41, 42
superspace, 10, 14, 33, 34, 35, 36, 37, 38, 39, 40
tNCS, 9, 10, 12, 18, 21, 22, 26, 27, 28, 29, 30, 34, 37, 41, 42, 43
translational and rotational non-crystallographic symmetry, 5, *See* tNCS
Translational non-crystallographic symmetry. *See* tNCS
translocation layer defects, 5

TRUNCATE. *See* Software
twinning, 5, 8, 11, 15, 18, 41, 43
Twinsolve. *See* Software
UCLA Twin Detection Server. *See* Software
XFEL, 5, 43
XPREP. *See* Software
