# Supplementary material for: Are the St John’s wort Hyp-1 superstructures different?
Source: Acta Crystallogr D Struct Biol. 2021 May 14;77(Pt 6):790–8. doi: 10.1107/S2059798321003740 (PMC8171068; doi:10.1107/S2059798321003740)
Supplement: Supplementary file 1 [file d-77-00790-sup1.zip › supp_2d_ex.pdf]

2D Supercell Expansion Example

In the paper “A non-mathematical introduction to the superspace description of modulated structures” (Wagner & Schonleber, 2009) the incommensurately modulated of structure of $C_{19}H_{27}NO_3Si$ is solved in superspace as well as a supercell approximation. The supercell approximation has 1/7 in $\mathbf{x_1}$ and 2/5 in $\mathbf{x_3}$. They provide a figure showing the supercell structures reordered in super space but don’t explain how. Superorder.m can be used to determine the reordering as shown below.

Supercell

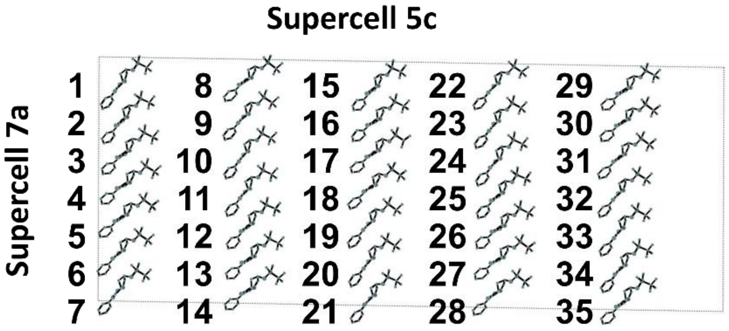

Superspace Ordering

$$x_4 = \begin{bmatrix} 1 & 32 & 28 & 17 & 13 & 2 & 33 & \dots \\ 22 & 18 & 14 & 3 & 34 & 23 & 19 & \dots \\ 8 & 4 & 35 & 24 & 20 & 9 & 5 & \dots \\ 29 & 25 & 21 & 10 & 6 & 30 & 26 & \dots \\ 15 & 11 & 7 & 31 & 27 & 16 & 12 & \end{bmatrix}$$

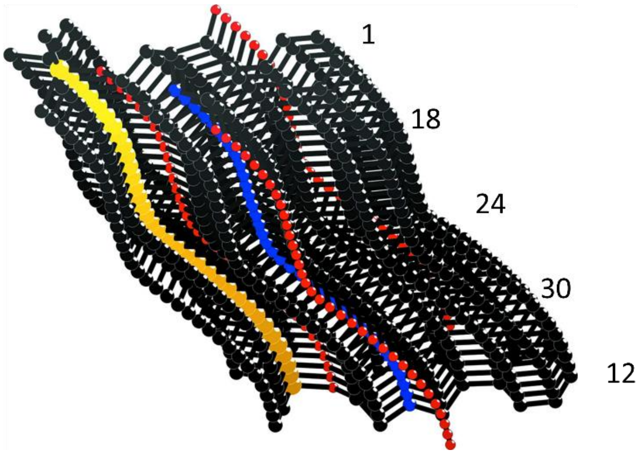

References

Wagner, T. & Schonleber, A. (2009). *Acta Crystallogr B* **65**, 249-268.
