# Supplementary material for: Are the St John’s wort Hyp-1 superstructures different?
Source: Acta Crystallogr D Struct Biol. 2021 May 14;77(Pt 6):790–8. doi: 10.1107/S2059798321003740 (PMC8171068; doi:10.1107/S2059798321003740)
Supplement: Supplementary file 1 [file d-77-00790-sup1.zip › supp_oc_mod.pdf]

Example of an AMF for a molecule that is occupationally modulated. In this case the occupational modulation is due to displacement modulations of the surrounding atoms that prevents the molecule from being able to fit in the binding pocket.

The empty circle represents the subcell where the molecule is missing.

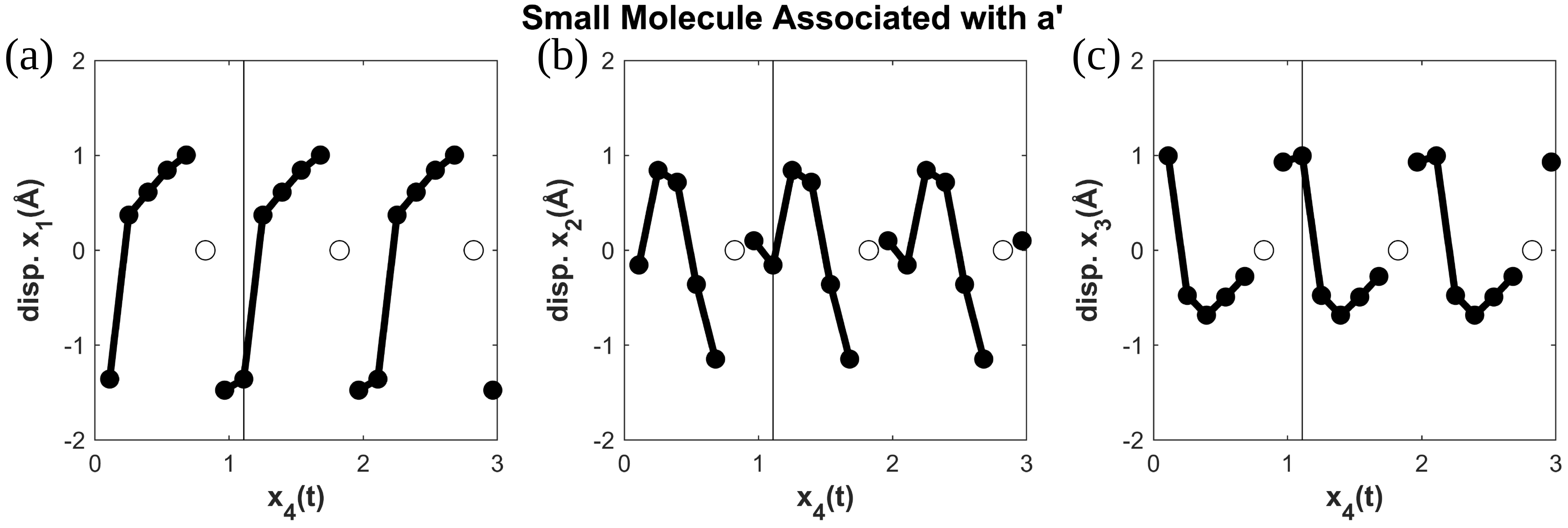

Supplemental: Occupational Modulation
