# Supplementary figures and images for: Are the St John’s wort Hyp-1 superstructures different?
Source: Acta Crystallogr D Struct Biol. 2021 May 14;77(Pt 6):790–8. doi: 10.1107/S2059798321003740 (PMC8171068; doi:10.1107/S2059798321003740)

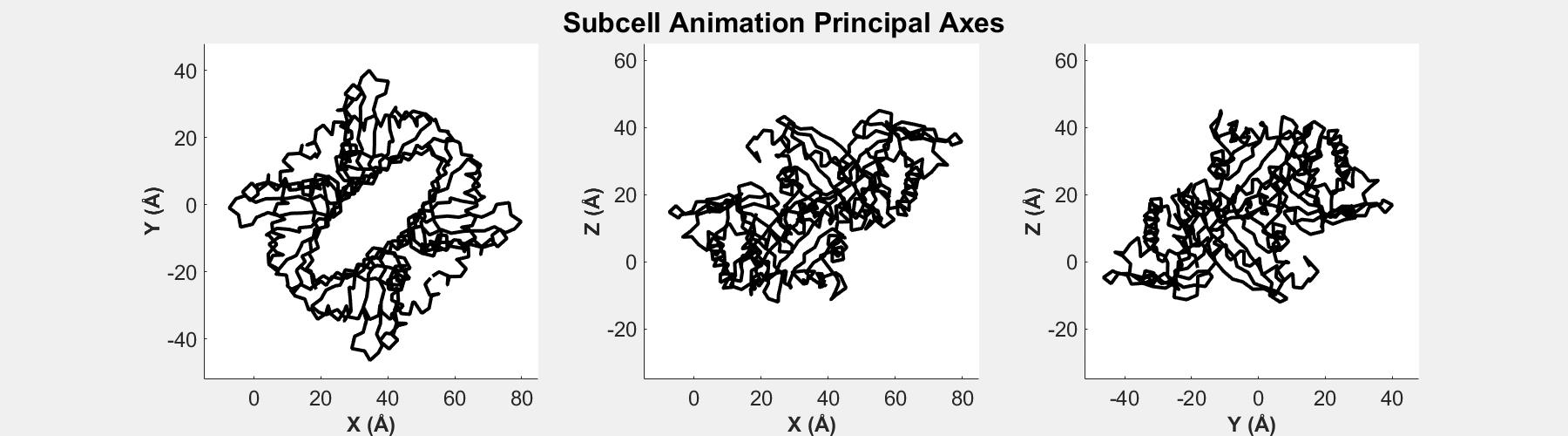

Supplement: Supplementary file 1 [file d-77-00790-sup1.zip › 4n3e_ans-3-7-3p.gif]

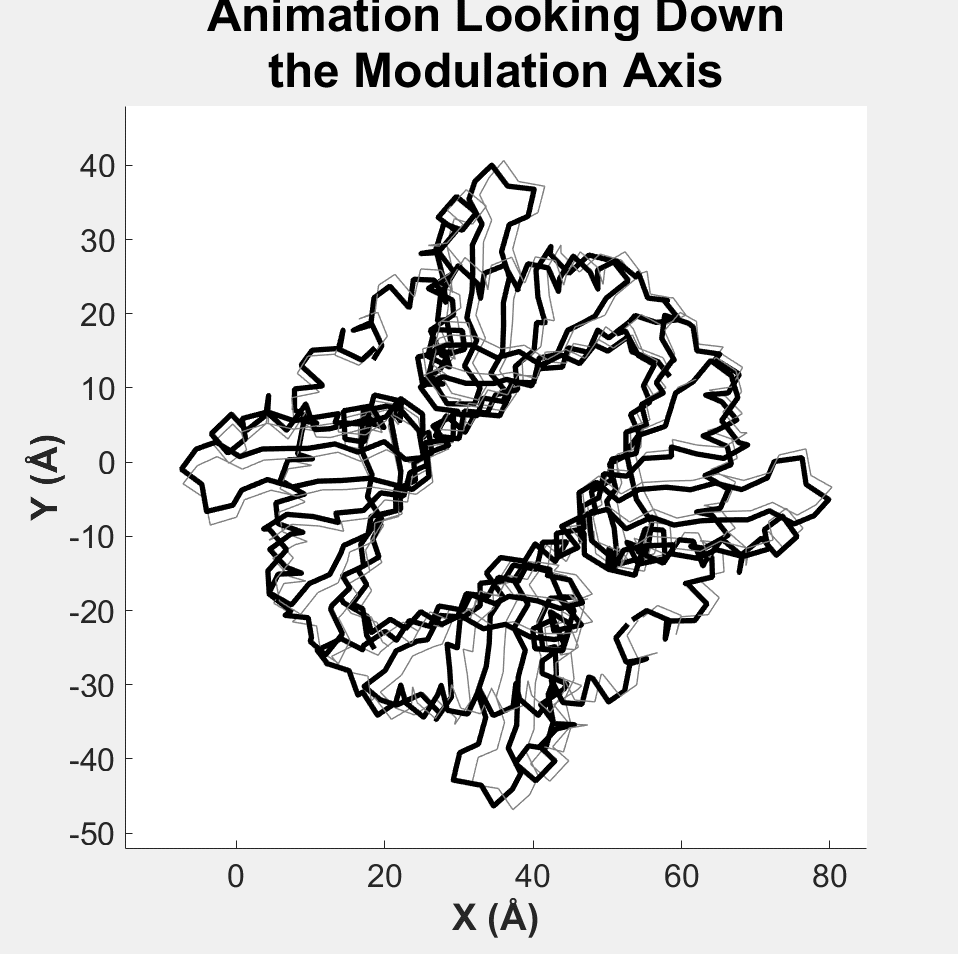

Supplement: Supplementary file 1 [file d-77-00790-sup1.zip › 4n3e_ans-3-7-ma.gif]

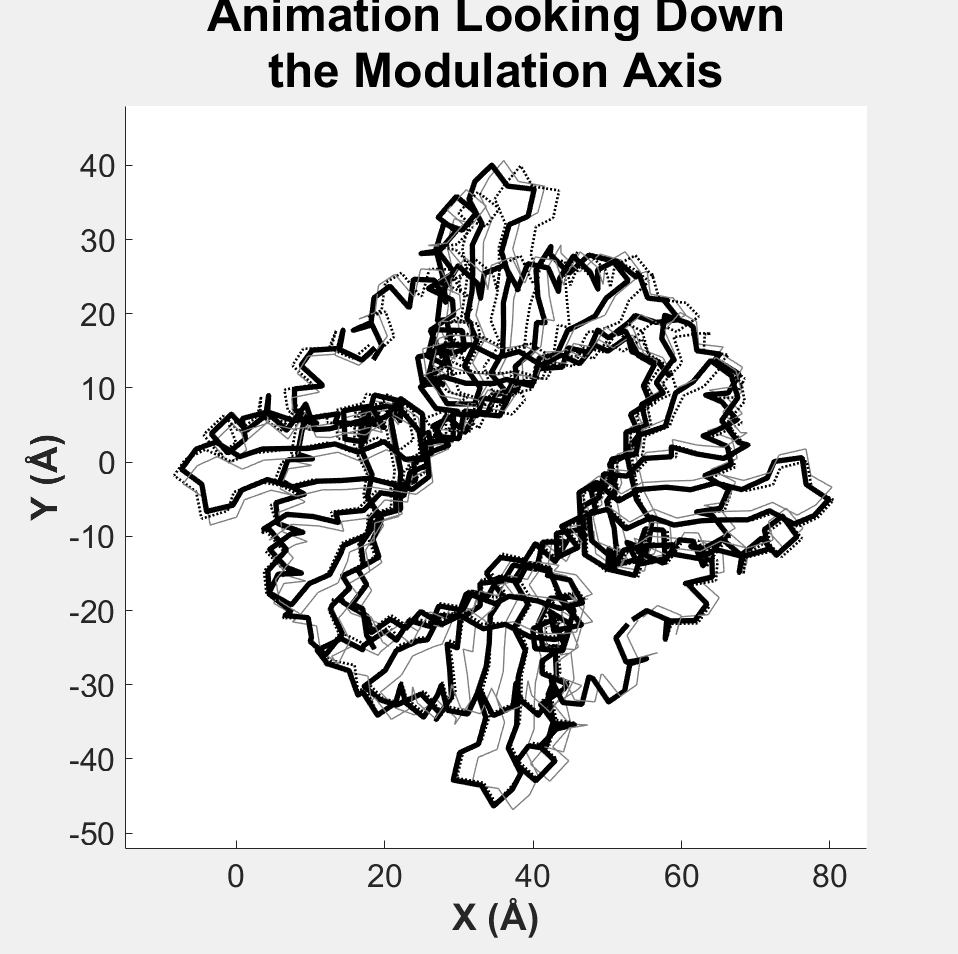

Supplement: Supplementary file 1 [file d-77-00790-sup1.zip › 4n3e_ans-3-7-mas.gif]

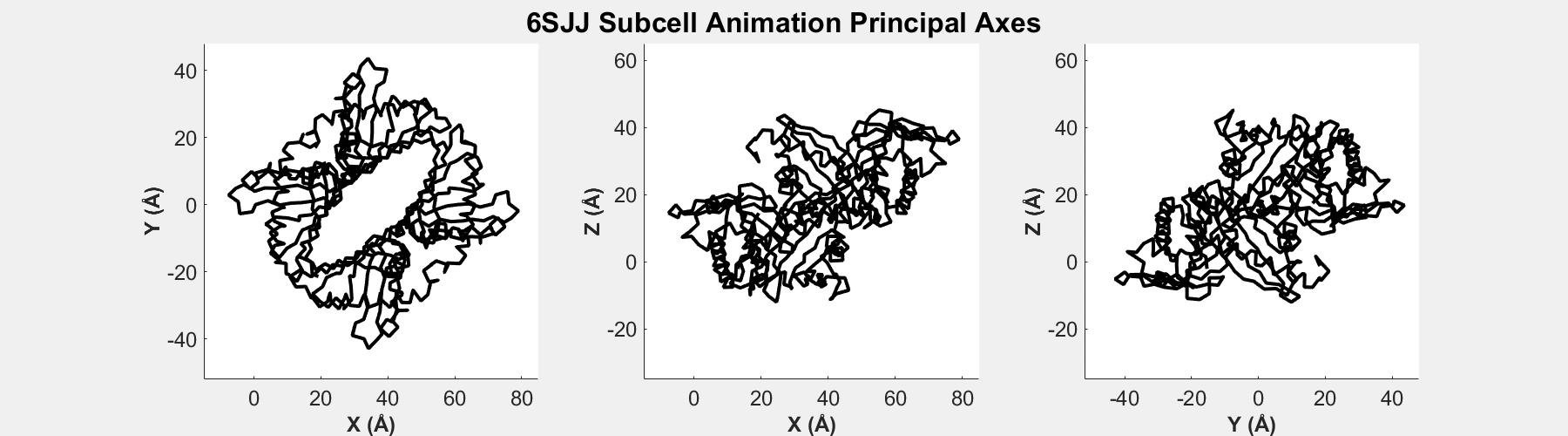

Supplement: Supplementary file 1 [file d-77-00790-sup1.zip › 6sjj_ans-4-9-3p.gif]

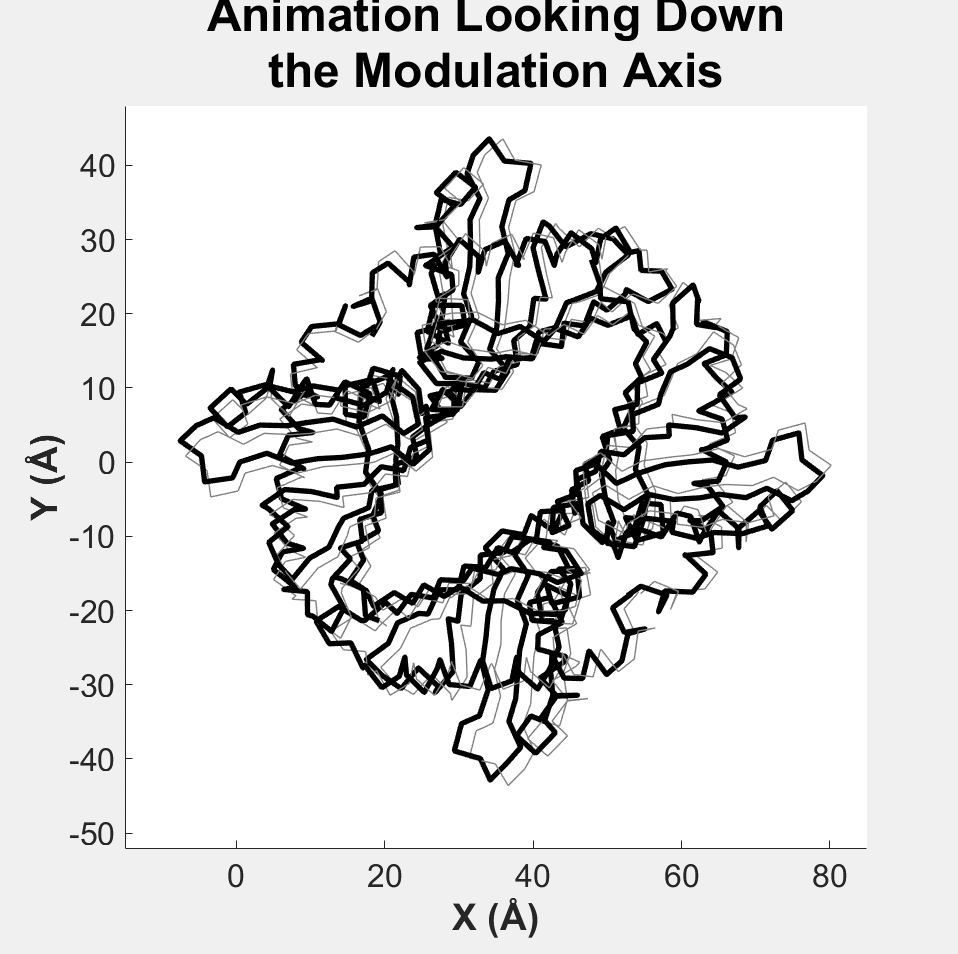

Supplement: Supplementary file 1 [file d-77-00790-sup1.zip › 6sjj_ans-4-9-ma.gif]

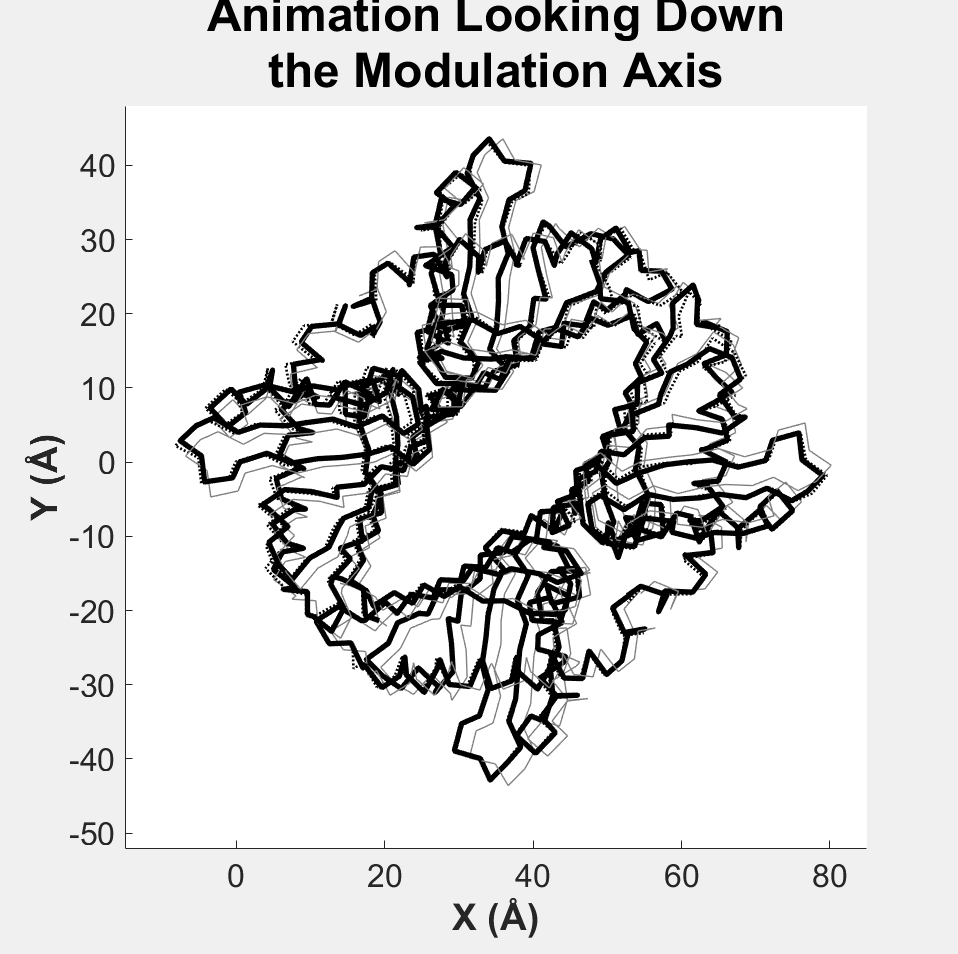

Supplement: Supplementary file 1 [file d-77-00790-sup1.zip › 6sjj_ans-4-9-mas.gif]
